# Manipulating concept spread using concept relationships

**DOI:** 10.1371/journal.pone.0199845

**Published:** 2018-06-28

**Authors:** James Archbold, Nathan Griffiths

**Affiliations:** Department of Computer Science, University of Warwick, Coventry, United Kingdom; Universidad Rey Juan Carlos, SPAIN

## Abstract

The propagation of concepts in a population of agents is a form of influence spread, which can be modelled as a cascade from a set of initially activated individuals. The study of such influence cascades, in particular the identification of influential individuals, has a wide range of applications including epidemic control, viral marketing and the study of social norms. In real-world environments there may be many concepts spreading and interacting. These interactions can affect the spread of a given concept, either *boosting* it and allowing it to spread further, or *inhibiting* it and limiting its capability to spread. Previous work does not consider how the interactions between concepts affect concept spread. Taking concept interactions into consideration allows for indirect concept manipulation, meaning that we can affect concepts we are not able to directly control. In this paper, we consider the problem of indirect concept manipulation, and propose heuristics for indirectly boosting or inhibiting concept spread in environments where concepts interact. We define a framework that allows for the interactions between any number of concepts to be represented, and present a heuristic that aims to identify important influence paths for a given target concept in order to manipulate its spread. We compare the performance of this heuristic, called maximum probable gain, against established heuristics for manipulating influence spread.

## 1 Introduction

In many environments it is possible for strategies, behaviours, knowledge or infections to spread within a population. The nature of propagation is determined by the interactions between individuals. Populations of autonomous entities are complex systems, meaning that the net effects of propagation are hard to predict or influence, despite being due to individual behaviour. Such propagation is a form of influence spread, which can be modelled as a cascade from a set of initially activated individuals [1].

Insight gained from understanding how to manipulate cascades in abstract populations has many applications, such as informing epidemic control, viral marketing, and understanding convention emergence in multi-agent systems. For example, characterising the spread of disease aids in identifying at risk groups, enabling containment efforts to be focused to avoid wider spread. Understanding how ideas propagate can inform viral marketing campaigns through identifying influential individuals. Other applications include the adoption of behaviours. It is known that behaviours, such as drinking and smoking, are often adopted by those who socialise with individuals that already partake in such behaviours. Furthermore, it has been shown that close acquaintances giving up smoking can encourage an individual to quit [2]. It may therefore be possible to identify individuals who, if they quit smoking, would result in the largest number of others quitting.

Modelling such a decision process can also inform an individual if they are being manipulated. For example, consider a competitive business such as finance, where a large factor in being successful is in the prediction of others’ behaviour. If a business can identify influential individuals and how their behaviours spread through a network, they may be able to identify when such an individual is taking an action as a strategy to influence others to take a similar action. Identifying this can allow for a business to avoid the manipulation tactic and make more informed decisions. The key enabler in all these examples is being able to identify the set of individuals who can help spread a behaviour, idea or product, or who can restrict future spreading (e.g. through their vaccination).

Several models have been developed to characterise influence spread [1, 3], along with techniques to maximise spread [4]. These models represent a population as a network, with individuals corresponding to the nodes, and edges representing the existence of an influential relationship from one individual to another. Opinions, behaviours, knowledge or diseases can be represented as concepts that spread along the edges of a network, with a chance to infect the nodes they encounter. Existing models of influence spread typically assume that only a single concept exists, or that concepts block the spread of other concepts, preventing an individual from activating multiple concepts [5, 6]. In real-world environments, individuals can have multiple concepts active simultaneously and, through concept interaction, a concept’s ability to spread can be affected. As such, in this paper we remove the assumption that concepts are blocking, allowing for more complex concept interactions.

Previous work on the interactions between spreading concepts has focused on epidemics and how interactions between diseases may affect the epidemic threshold of a network [7]. However, there has been little consideration of concept interactions to help improve the spread of a given *target concept*, in situations where we cannot directly manipulate the spread of that target concept. Consider, for example, a disease spreading through a network. While it may not be possible to affect the disease directly, its spread can be inhibited through the spread of knowledge and the use of vaccination, both of which can also be modelled as concepts within a network. The interactions between such concepts may be positive or negative, aiding or hindering spreading respectively.

In previous work, we developed a heuristic that aims to maximise the spread of a chosen concept in an environment where concepts interact. This involved selecting seed nodes to avoid other, inhibiting, concepts while increasing the chance to encounter boosting concepts [8]. However that work, along with most previous work on influence spread, assumed that we can select seed nodes for the target concept we wish to maximise. There has been little consideration of scenarios where this is not the case. When we cannot control the target concept that we aim to manipulate, we must instead indirectly affect its spread through a secondary controllable concept. It is this problem of *indirect* concept manipulation that is the focus of this paper.

Concept interactions facilitate the manipulation of a concept that cannot be directly controlled. Consider opinions spreading within a social network. It is extremely difficult to guarantee an individual’s adoption of a chosen opinion, instead we may consider exposing an individual to particular information or news stories that makes them more, or less, likely to adopt that opinion themselves. This may be of use in encouraging loyalty to a given brand or promoting particular social habits within a group. If, for example, we wish to spread the opinion that smoking is detrimental to health, we may choose to expose certain individuals to information on the health risks of smoking, making them more likely to agree when a friend shares a negative opinion on smoking. The use of a secondary concept to boost the spread of a given target concept is known as the *indirect influence maximisation problem*.

Alternatively, we may wish to indirectly inhibit the spread of a detrimental concept such as a disease or rumour. A potential approach to minimising the spread of a target concept is to expose a selected group of individuals to a secondary inhibiting concept. This is known as the *indirect influence limitation problem* [9]. Previous investigations into minimising influence have focused on finding nodes present on a high number of shortest paths [10], or nodes that connect communities [11]. In environments where concepts are blocking, selecting such nodes to block concept spread prevents the undesirable concept from utilising the most influential network paths. However, when concepts are not blocking but instead merely lower the chance of spreading, such methods are less effective. If a node is on many shortest paths, but is not near to the start of the target concept cascade, it is unlikely to encounter the target concept, and so its selection is unlikely to help limit the concept spread. As such both a node’s expected gain for the target concept and the likelihood of activating that target concept, must be considered when evaluating a node’s suitability for influence limitation.

In this paper, we investigate the use of a secondary concept to affect, positively or negatively, the spread of a target concept. We present a framework for representing concept interaction, and discuss the differences between indirect concept spread manipulation and the more widely studied influence maximisation problem. With those differences in mind, we propose the maximum probable gain (MPG) heuristic and evaluate its effectiveness in both the indirect influence maximisation and indirect influence limitation problems. A subset of our experiments for the influence limitation problem, namely the performance of MPG and other heuristics in small-world, scale-free and real-world networks for the ICM has been previously published in [9].

## 2 Related work

Previously, there have been many proposed models of influence spread and corresponding approaches to the influence maximisation problem. Here, we highlight and discuss important models and methods, and introduce previous work that considers multiple concepts or uses concept interactions to affect the spread of a specific target concept.

### 2.1 The influence maximisation problem

The influence maximisation problem is that of finding a set of *k* nodes, referred to as the seed set, that will maximise the spread of a concept, for a given network. Kempe *et al*. were among the first to describe this problem, in addition to methods of modelling the propagation of a concept [1]. Since then, many influence propagation models have been proposed in the literature [3, 12, 13]. Two of the most widely discussed, in terms of modelling influence spread, are the Independent Cascade Model (ICM) and the Linear Threshold Model (LTM).

In the ICM a node has a single chance on activation to activate each inactive neighbour with some probability, *p* [3]. Alternatively, in the LTM a node is influenced by each of its neighbours to varying degrees, as defined by the edge weights in the network [1]. Each node *v* has a threshold *θ*_*v*_, and when the sum of the weights of *v*’s active neighbours exceeds *θ*_*v*_, *v* becomes active. Influence cascades have also been utilised to model epidemic spread [7, 14], leading to the Susceptible Infected Susceptible (SIS) and Susceptible Infected Recovered (SIR) [12, 13] models, which take a probabilistic approach to the ICM, allowing for deactivation. These models allow for a node to deactivate a concept, after which the node can reactivate the concept later, as in the SIS model, or the node can never reactivate the concept even if exposed to it again, as in the SIR model. The ICM can be viewed as an instantiation of the SIR model where a node deactivates the concept after only a single time step.

Influence related problems typically focus on the selection of a ‘seed set’. This is a set of nodes that are selected to activate a concept and initiate that concept’s spread through a network. Ideally, a seed set of size *k* will contain the *k* most influential nodes within the network, however identifying these nodes is a challenging problem. A greedy approach is often effective in approximating the optimal solution, due to the sub-modularity of seed set selection [1, 11]. Hill climbing can be used to select the node that provides the largest incremental increase to the performance of the current seed set, with Kempe *et al*. showing that this approach has a performance guarantee of slightly above 63% of the optimal solution [1]. Others have developed algorithms based on hill climbing, such as the Cost Effective Lazy Forward (CELF) selection algorithm proposed by Leskovic *et al*. [14], which evaluates the improvement in reward from including a node in the current seed set and adds the node that maximises that improvement. However, calculating the reward of adding a node involves simulating a number of cascades and recording the number of activations achieved by a given seed set. For real-world problem sizes, this approach is often intractable, since it has a time complexity of $O(n^{3})$ or higher [15]. Despite this, hill climbing is often used as a baseline for evaluating new heuristics for maximising influence spread. Heuristics, such as degree discount for the ICM, allow for the efficient approximation of a node’s performance improvement [4].

Degree discount has been shown to be an effective method of influence maximisation, comparable in performance to the greedy algorithm [4]. It calculates the expected activations gained from the inclusion of a particular node in the seed set. When a node is selected as a seed, its neighbours have a chance to be activated in the first round of a cascade. As such, the expected gain of those neighbours is lowered. Nodes are initially ranked by degree, and when a node is added to the seed set its neighbours have their degree set to *d*_*v*_ − 2*t*_*v*_ − (*d*_*v*_ − *t*_*v*_) * *t*_*v*_ * *p*, where *d*_*v*_ is the original degree, *t*_*v*_ is the number of neighbours in the seed set and *p* is the probability of infection. This calculation is based on the expected benefit of such nodes (details of its derivation can be found in [4]).

Other methods include the consideration of a *k*-core decomposition [16]. To begin, all nodes with only a single edge are removed, along with their edges, and then any nodes now left with only one edge are also removed. This continues until there are only nodes with two or more edges in the network. The removed nodes, along with any edges that connect those removed nodes, are the first k-shell. By repeating this, we can identify the nodes that make up the core of the network as ones with a high k-shell value. This method has been debated, with some claiming that it is not as effective as degree-based selection [17, 18] and that nodes in the same k-shell can have very different levels of influence and so a method of ranking nodes in the same k-shell is needed [19].

Some greedy approaches can be made tractable, such as the Greedy Viral Stopper (GSV) algorithm, for environments in which ‘correct’ and ‘incorrect’ information propagates, with the correct information superseding incorrect information [20]. While intractable in the general case, the GSV algorithm can be performed on individual communities in a network [21]. By combining those individual solutions, a full network solution can be achieved.

### 2.2 Influence models with multiple concepts

The majority of existing influence spread models focus on single cascades. However, in real-world environments, there may be many concepts simultaneously spreading. This has led to the consideration of multiple influence cascades [5, 6]. Such work typically assumes that cascades are *blocking*, meaning that nodes activated by one cascade cannot be activated by another. Additionally, most formulations assume that an environment contains only two concepts, when in reality there could be many more, all of which interact.

If concepts are not blocking, then how they interact must be modelled. Sanz *et al*. developed a multi-layer network model in which each concept spreads on a different layer, but nodes can activate more than one concept at a time [7]. When multiple concepts are active on the same node, they can boost or inhibit each other’s ability to spread. The idea of boosting and inhibiting the spread of a concept has been explored by others [9], including the effects this can have on concept interactions. Newman and Ferrario consider a model where an epidemic can only spread through nodes that have been infected by a previous epidemic [22]. In the work of Myers and Leskovec the concepts an agent chooses to adopt are affected by all the concepts they have previously been exposed to (regardless of adoption) [23]. Such models typically focus on concepts that are similar to each other, such as diseases, but other factors, such as the information available on that disease or the availability of vaccinations can also affect spread [10].

Related, but semantically different, concepts can also interact. Wang *et al*. demonstrated, with multi-layer networks, the relationship between a disease and information about that disease. As the disease propagates, it increases the spread of information about it, which in turn allows people to make informed decisions and decrease the spreading rate of the original disease [24]. Multi-layer networks can model multiple concepts by restricting each concept to its own layer, allowing for the modelling of transmission methods distinct to each concept [25]. For example, a concept spread by hand-to-hand contact will spread differently than one that utilises word-of-mouth. Multi-layer networks have also been utilised to facilitate the analysis of the effectiveness of introducing an immunising concept to a network that contains a spreading disease [26].

An individual’s opinions can affect the concepts they adopt or spread. These opinions can be represented through network and node attributes, as in the adaptation of the LTM proposed by Kaur and He [27]. Each edge is associated with two separate influence strengths, representing a positive and negative opinion respectively. A node has both a positive and negative threshold, and will activate the opinion that first has its corresponding threshold exceeded. Nodes with high positive influence can be selected to block the negative opinion from spreading further. Stitch *et al*. present a method of representing opinions by assigning an attitude score to individual nodes [28]. Nodes with a high attitude score are more likely to spread negative word-of-mouth, even if they have been mostly exposed to positive opinions, and vice versa for nodes with low attitude scores. Both of these models again assume that concepts are blocking.

A further factor that has been shown to impact concept spread is the general behaviour of a population. Perra *et al*. present a model that changes the infectiousness of an epidemic over time to reflect behaviour [29]. Other approaches include modifying network connections to simulate fear [30], introducing an additional edge weight to represent social connections [31], and the use of game theory to model strategies that can change as the epidemic spreads [32]. The idea of responsiveness was introduced by Kiss *et al*., with responsive individuals more likely to avoid infection or be treated quicker, reducing the spread of a disease [33].

### 2.3 Utilising concept interaction

There has been little consideration of indirectly maximising the spread of a concept, with the majority of previous work that does consider concept interactions focusing on influence limitation. Fan *et al*. propose using nodes that connect one community within a network to another, known as bridge ends, to block an undesirable concept from reaching large communities within the network [11]. Li *et al*. select nodes for immunization that will protect the bridge ends themselves, further distancing an undesired concept from other communities within the network [34]. Other methods have also been proposed, such as removing central nodes, partitioning the network and making it more difficult for a disease to spread [35]. While protecting bridge ends can limit the spread of a concept, the approach becomes less effective when concept, and therefore path, blocking cannot be guaranteed. The betweenness of nodes can also be utilised as an indicator of a node’s potential to limit a concept’s spread, but is computationally expensive to calculate [10].

Kotnis and Kuri propose a solution where individuals can be trained, at a cost based on their degree and the quality of training, to be better at deciding whether information is a rumour [36]. For a given budget, having more low quality trained individuals yields better results than having fewer individuals with higher quality training. While Kotnis and Kuri assume a single cascade, they discuss the possibility of other messages affecting the spread of a rumour.

A related problem, selecting a group of nodes and improving their ability to spread a target concept, is discussed by Liontis and Pitoura [37]. They present the MoBoo heuristic, which selects nodes to become able to boost the probability of spreading a target concept by evaluating the possible gain from selecting each node.

To estimate the gain of a node, MoBoo first finds the likely paths that the target concept will travel through. For each node with the target concept active, MoBoo constructs a tree of paths that start from that node. With these trees in place, MoBoo then constructs λ independent paths, where typically λ = 2 in order to maintain a reasonable runtime. For each node, *v*, MoBoo selects the top λ paths where the only nodes shared are the end node, *v*, and possibly the starting node. These paths are referred to as *μ*^*i*^, where *i* is the rank of the path. Paths are ranked by their propagation probability and so MoBoo selects the λ paths that are most likely to activate *v*.

Using the propagation probability of these paths, the *activation probability* of a node, *ap*(*v*) can be calculated. For λ = 2:

$$ap\left(v\right)=1-(\left(1-P\left(\mu^{1}\left(v\right)\right)\right)\times \left(1-P\left(\mu^{2}\left(v\right)\right)\right))$$ (1)

where *P*(*μ*^*i*^(*v*)) is the probability of *v* to be activated from independent path *i*.

These activation probabilities are used to calculate the gain for each node. The gain for a node, *v*, assumes that *v* has the boosted probability to spread the target concept to its neighbours and is calculated as:

$$g\left(v\right)=\sum_{u\in Out(v)}(\frac{p_{v,u}^{\prime }}{p_{v,u}}-1)\sum_{w\;descendant\;of\;u}ap\left(w\right)$$ (2)

where *Out*(*v*) is the set of nodes that are the children of *v* in any independent path *μ*, *p*_*v*, *u*_ is the probability of the concept spreading from node *v* to *u* and $p_{v,u}^{\prime }=p_{v,u}+b$, with *b* being the improvement to spreading probability gained by a node when it is selected to become a boosting node.

In each round, we select the node, *v*, with the highest gain, *g*(*v*), and update the required paths. This is repeated until we have selected the desired number of nodes. Full details of the derivation of this approach can be found in [37]. The approach is based on the Prefix excluding Maximum Influence in-Arborescence (PMIA) algorithm for influence spread, which considers the nodes likely to activate a concept, and the expected activations gained by the influence of those nodes [38].

The MoBoo algorithm focuses on boosting concepts, but the potential influence of a node may also be of use when considering an inhibiting concept. Targeting high influence paths with a boosting concept should provide a larger performance increase for the target concept than a low influence path. Similarly, limiting the potential of a high influence path may prove more damaging to a target concept than targeting a low influence path.

Budak *et al*. propose the highest infectees heuristic that, for environments with ‘good’ and ‘bad’ cascades, gives results comparable to greedy hill climbing [39]. This heuristic assumes knowledge of the seed set for the ‘bad’ cascade, and simulates a large number of cascades using that seed set. Nodes are ranked by the number of simulations in which they became infected with the ‘bad’ cascade, and are selected as seeds for the ‘good’ cascade in descending order. While the aim of this work is to limit the spread of the ‘bad’ cascade, it does so through maximising the spread of a ‘good’ cascade. Therefore this may also be an applicable approach for both boosting and inhibiting a target concept through a secondary concept, in order to increase the number of concept interactions.

Rather than selecting nodes to block a concept’s spread, we may also consider modifying the edges in a network. Li *et al*. proposed reducing the edge weight to limit the rate of transmission [40]. An edge’s weight is expressed as a function of the degree of its two end points, and the rate of transmission between two connected nodes is proportional to the weight of the edge between them. The use of inflammation immunisation, which reduces edge weights by a chosen factor, is shown to be effective in this model and may translate well to real-world scenarios. Notably, the reduction of edge weights lowers the transmission weight but does not compromise network efficiency. Conversely, the bond percolation approach suggested by Kimura *et al*. curbs the spread of an infection but, by removing links, damages the network structure and in turn, the ability of the network to transmit other concepts [41].

Overall, we see that most previous work involving multiple cascades assumes that concepts block. This leads to a focus on competitive influence spread, where the aim is to maximise the spread of one cascade over another, where we directly choose the seed nodes for the concept we wish to maximise. Through more complex concept interactions, we can move beyond competitive influence and consider indirect influence manipulation. The work of Liontis and Pitoura [37] is one of the few approaches to consider indirect influence maximisation, although the ability to boost a concept’s spread cannot be spread itself.

### 2.4 Real-world experiments

The studies discussed above all rely on empirical results from simulations. This is due to the difficulty of performing real-world analysis on the spread of influence. In particular, there is not always a precise definition of what constitutes an individual being ‘influenced’. In studies involving the social media site Twitter, re-tweeting and using hash tags have been used to signify that a person has been influenced, but this is not necessarily a reliable method [42]. If we cannot confidently state that an individual has been influenced, tracking a cascade is difficult.

Furthermore, having full knowledge of a social network is difficult. Presently, the majority of influence spread studies assume that we have knowledge of the full network and do not account for any possible hidden factors. The requirement of full knowledge for such studies makes translation to real-world scenarios difficult.

However, this does not mean that real-world studies have not been performed. There has been recent work which has considered spreading information in a local network of homeless youth that frequent particular shelters [43, 44]. This work focuses on the uncertainty problems, but demonstrates that influence spread studies can be performed in the real-world, with some tangible effect, and that there is value in empirical simulations since the results can be subsequently applied in real-world environments.

## 3 Indirect concept spread

Individuals, or nodes, in real-world networks are typically capable of activating many concepts at once, representing a wide range of ideas, opinions and attributes. Furthermore, concepts may be related to each other and have the ability to affect each other’s spread. As a result, the concepts active on a node may increase or decrease the likelihood that the node will activate other concepts in the future.

Previous work on limiting the spread of a concept typically assumes that concepts are blocking [11, 27, 28, 34]. However, Sanz *et al*. presented a model for the interactions of two diseases spreading through a network, where an individual infected with one disease is more likely to be infected with the other [7]. This demonstrates the value of considering concept interaction, and highlights that such interactions could be used to indirectly affect a target concept. We adopt the view of Sanz *et al*., in assuming that concepts are not blocking.

Utilising the interactions between concepts allows for the manipulation of concepts that may not be directly controlled. For example, we could encourage the spread of an opinion through supporting information, or slow the spread of a disease with targeted vaccination. At the start of an influence cascade, some nodes will have concepts active on them, which can then spread to other nodes. The initial set of nodes that activate a concept is known as the seed set for that concept. When attempting to manipulate the spread of a concept indirectly, we consider two different types of concept, the target concept and the controllable concept:

*Target Concept*: A concept for which we cannot directly select seed nodes within the network but wish to manipulate its spread.
*Controllable Concept*: A concept for which we can directly place seed nodes within the network, that interacts with the target concept and affects the target concept’s spread.

Indirectly controlling the spread of a concept can be divided into two different problems. The *indirect influence maximisation problem* aims to identify a seed set of size *k* for a boosting controllable concept that will maximise the spread of a target concept. Conversely, the *indirect influence limitation problem* aims to identify a seed set of size *k* for an inhibiting controllable concept that will minimise the spread of a given target concept. In both cases, the problem becomes how best to place seed nodes for a secondary concept to maximise our impact on the spread of the target concept, either positively or negatively.

When the objective is to leverage a controllable concept to indirectly manipulate a different concept, we must reconsider how we value nodes. In the traditional influence maximisation problem, nodes are valued on their ability to facilitate the spread of the target concept to the highest number of neighbours possible. For indirect concept manipulation, we must consider whether a selected node will help facilitate concept interactions, and the extent to which it will impact the spread of the target concept. With indirect concept manipulation, the final spread of the controllable concept is unimportant, since the concern is maximising the controllable concept’s effect on the target concept. This does not mean, however, that maximising the spread of the controllable concept is not a viable approach. If the controllable concept is spread widely throughout the network, then it will be more likely to interact with the target concept and thus impact its ability to spread.

An alternative simple strategy is to select all the nodes that currently have the target concept active. Selecting nodes that are actively spreading the target concept will naturally result in guaranteed interactions between the target and controllable concepts, contributing to the goal of indirect influence manipulation. However, we may not have the budget to target every node that is currently spreading the target concept, especially if the two concepts are not introduced into the network at the same time and so the target concept has already spread. Alternatively, we may also be able to select more seeds for the controllable concept than there are nodes currently spreading the target concept. Therefore, we would require a method to identify which nodes with the target concept active are the most valuable to select, and a method to evaluate the value of nodes without the target concept active.

Instead, we may consider the paths a concept is most likely to travel. Analysing the likely paths a concept will travel from a given node, and evaluating the activations expected as a result of travelling those paths, allows for nodes to be ranked in terms of their contribution to the spread of a chosen concept. Nodes that have a higher number of expected activations are naturally more valuable to the target concept and, through the use of concept interaction, the number of expected activations can be manipulated. Attempting to evaluate the full set of paths from every node within in a network is prohibitively computationally expensive. However, the search space can be restricted to consider only paths above a given probability of being taken, allowing for the tractable estimation of the expected activations of a node.

In the ICM, each successive round of a cascade typically infects fewer nodes than the previous round due to the relatively low probability of infection. Therefore, a concept performs the majority of its spreading in the early time steps of a cascade and so will be more affected by concept interactions in the early stages than at a later time. Furthermore, the higher the expected gain of a node, the greater its impact on the spread of the target concept. Naturally, if the target concept becomes active on a node with high expected gain, the concept can spread further and be more likely to encounter other nodes with a high expected gain. If we wish to boost the spread of a target concept, then targeting nodes with a high expected gain increases their impact even further. If the goal is to inhibit a target concept’s spread, targeting these nodes will result in a higher number of lost activations than targeting nodes with a lower expected gain. As such, for both the indirect influence maximisation problem and the indirect influence limitation problem, targeting nodes with a high expected gain could prove effective.

## 4 Concept interaction framework

To model complex concept interactions, we propose a framework based on the model presented by Sanz *et al*. [7]. In this paper, we focus on the ICM and LTM, due to their widespread use in previous work [1, 3, 4, 14]. In the ICM, newly activated nodes have a single opportunity to spread a concept to each of their neighbours, with a probability, *p*, of success [3]. Once a node activates a concept, it remains activated permanently, but cannot attempt to spread the concept after its initial opportunity. In the LTM, every node has a threshold that incoming influence must exceed before they activate a concept and begin exerting influence on their neighbours [1]. Again, once a node is activated it remains activated permanently but, unlike in the ICM, a node continually exerts influence on its neighbours. This means that a node’s neighbours may activate a concept over several time steps, until their collective influence causes the node to activate the concept. In both models, influence travels from one node to another through interactions. We call the node that the concept or influence is travelling from the infector node and the node that the concept or influence is travelling to the receiver node. For example, in the ICM a newly activated node is the infector and may spread a concept to each its neighbours, which are receivers. In this framework, the concepts already active on a receiver affect the probability of an additional concept being adopted by that receiver. Furthermore, the concepts active on an infector affect the probability of those concepts spreading to its neighbours. Although we define propagation and influence strength in terms of the ICM and LTM, our concept interaction framework is generally applicable and can represent a range of spreading dynamics. Furthermore, while we define the interactions between only two concepts, we briefly discuss how this framework can be extended to an arbitrary number of interacting concepts. Table 1 summarises the notation used to described the framework.

**Table 1:** Notation used to denote the concept interaction framework.

Parameter	Values
$N_{v}^{i}$	The set of incoming neighbours for node *v*.
$N_{v}^{o}$	The set of outgoing neighbours for node *v*.
*I*_*v*,*u*_(*c*)	The strength of influence exerted by node *v* on node *u* relating to concept *c*.
*CI*_*v*,*u*_(*c*)	The contextual influence exerted by node *v* on node *u* relating to concept *c*. This is calculated by scaling *I*_*v*,*u*_(*c*) appropriately.

### 4.1 Environment

We represent an environment by a set of nodes situated in a network. Each node, *v*, has a set of incoming neighbours, $N_{v}^{i}$, and outgoing neighbours, $N_{v}^{o}$, where nodes in $N_{v}^{i}$ can influence *v*, and *v* can influence nodes in $N_{v}^{o}$. These sets are not necessarily disjoint and may be equivalent in some environments, allowing both directed and undirected graphs to be represented.

### 4.2 Influence Spread

A concept spreads through a network during interactions between nodes. During these interactions, a concept can spread from the infector node to the receiver node dependent on the strength of the influence being exerted by the infector onto the receiver. *I*_*v*,*u*_(*c*) represents the influence strength exerted by node *v* onto node *u* with respect to concept *c*. The value of *I*_*v*,*u*_(*c*) is defined based on the influence spread model that is used.

The ICM emulates word-of-mouth propagation, with concepts being spread from node to node based on single interactions. As discussed previously, a concept successfully spreads from an infector to a receiver with a given probability *p*, and each infector-receiver pair can interact only once per concept. We define *I*_*v*,*u*_(*c*) = *p* for any pair of nodes such that $v\in N_{u}^{i}$ and *p* is the chance of infection. In each time step, each node that activated a concept in the previous step can attempt to spread that concept to each of its neighbours.

The LTM emulates the idea of group influence, with nodes activating a concept based on the number of their neighbours that have already activated that concept. Each node, *n*, has a threshold, *T*_*n*_, that represents the total incoming influence required for a node to activate a concept. A pair of nodes is connected by a directed edge, with a given weight, *w*_*v*,*u*_, that represents the strength of influence from the source node *v* to the destination node *u*. In the original, single concept, LTM, a node activates the concept once the sum of incoming weights, *w*_*v*,*u*_ from neighbours with the concept active exceeds its own threshold.

When there are multiple concepts, a node *n* will have an individual threshold, $T_{n}^{c}$, for each concept, *c*. Similarly, edges will have individual weights, $w_{v,u}^{c}$, for each concept, leading to an environment that is equivalent to a multi-layer network. Each concept is then activated on a node in a similar way to the original LTM. When the total incoming influence for a concept *c* exceeds a node’s threshold for *c*, that node activates *c*. For a given concept *c*, we define $I_{v,u}\left(c\right)=min\left(1,w_{v,u}^{c}/T_{u}^{c}\right)$. We divide the weight of the edge between the two nodes by the receiver node’s threshold, so that influence strength represents the proportion of the receiver node’s threshold that is matched by the edge weight. Accounting for the threshold of the receiver allows for a relative measure of influence strength, which can be better compared to other relationships within the network. We limit *I*_*v*,*u*_(*c*) to a maximum value of 1 since that signifies a guaranteed activation.

### 4.3 Concept interaction

When nodes interact, the spread of a concept can be affected by other concepts active on both the infector and the receiver. In this model, this effect is represented by scaling the influence strength that an infector *v* can exert on a receiver *u* regarding concept *c*. The resulting value is referred to as the contextual influence and is represented by *CI*_*v*,*u*_(*c*).

**Fig 1:**
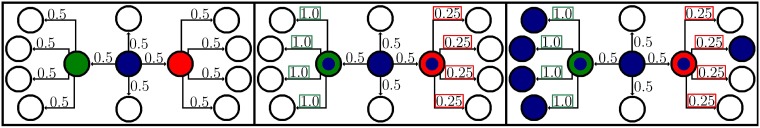
Illustration of a target concept (blue) spreading through a network, being affected by a boosting concept (green) on the left and an inhibiting concept (red) on the right. Note how the edge weights change when the target concept interacts with the boosting and inhibiting concept, increasing on the left from an interaction with the boosting concept and decreasing on the right from an interaction with the inhibiting concept.

The definition of *CI*_*v*,*u*_(*c*) is dependent on the use case, in particular the number of concepts that can interact within the model. For our case, where we have only a target concept, *t*, and a secondary controllable concept, *s*, we define *CI*_*v*,*u*_(*t*) as follows:

$$CI_{v,u}\left(t\right)=\{\begin{array}{cc} min(1,I_{v,u}\left(t\right)) & \text{if}\;s\;\text{is}\;\text{not}\;\text{active}\;\text{on}\;\text{either}\;v\;\text{or}\;u\\ min(1,I_{v,u}\left(t\right)*r) & \text{if}\;s\;\text{is}\;\text{active}\;\text{on}\;\text{one}\;\text{of}\;v\;\text{or}\;u\\ min(1,I_{v,u}\left(t\right)*r^{2}) & \text{if}\;s\;\text{is}\;\text{active}\;\text{on}\;\text{both}\;v\;\text{and}\;u \end{array}$$ (3)

where *r* ∈ [0, ∞) is a feature of the environment that represents the extent to which concept *s* affects concept *t*. If *r* < 1, then *s* decreases the ability of *t* to spread, and the relationship is said to be inhibiting. If *r* > 1, the relationship is said to be boosting as *s* increases the ability of *t* to spread. If *r* = 1 then *s* has no impact on the ability of *t* to spread. As discussed previously, the concepts active on an infector can affect the concepts that spread from it, and the concepts active on a receiver affect the concepts the receiver will adopt. As such, we consider the context of both nodes when evaluating the strength of the contextual influence. Note that we limit the contextual influence value to a maximum of 1 as, in both the ICM and LTM, that means the receiver is guaranteed to activate the target concept. The possible effects of concept interaction are illustrated in Fig 1, where we see a boosting concept increase the number of neighbours activated and an inhibiting concept decrease the number of neighbours activated.

The concept interaction model is designed to be extensible and allow for any number of concepts to spread within a network and interact. If there are more than two concepts that can interact, each relationship must be defined. In particular, this requires defining how each concept affects the spread of others when active on the infector and when active on the receiver. Each of these situations can be defined using a value for *r*. When a concept, *c*, spreads, *I*_*v*,*u*_(*c*) is then scaled by the summation of the effects of each concept active on the infector and a summation of the effects of each concept active on the receiver. While in this work we do not fully utilise the wider functionality of this model, the used to run the simulations has been designed to allow for the most general case and is obtainable from: https://github.com/JamesArchbold/ConceptInteraction.

## 5 Indirect concept manipulation

The influence maximisation problem has been well studied, leading to a variety of heuristics that aim to approximate an optimal solution. However, there may be situations where we do not have direct control of the concept we wish to manipulate, as discussed in Section 3. Concept interactions provide an opportunity for us to indirectly manipulate the spread of another concept, allowing us to tackle a wider range of problems when compared to assuming blocking concepts.

In this section, we propose the maximum potential gain (MPG) heuristic to address the indirect influence maximisation and indirect influence limitation problems in environments with concept interaction. Algorithm 1 defines the MPG heuristic and in this section we describe its operation in detail. A summary of the notation used in our description can be found in Table 2. Previous techniques that have been developed for environments where concepts block are less effective when dealing with concept interaction, as it becomes impossible to guarantee that a concept can be prevented from spreading. As such, new techniques are needed for indirect influence manipulation, leading to the development of MPG.

**Table 2:** Notation used to describe MPG.

Parameter	Values
*t*	The target concept.
*S*_*t*_	The set of nodes that can spread the target concept in the next time step.
*MIP*(*v*, *u*)	The most influential path from *v* to *u* for *t*, i.e. the path with the highest amount of influence originating at *v* and ending at *u*.
*θ*	The minimum influence value for a MIP(v,u) to contribute to either ap(u) or E(v).
*ap*(*v*)	The activation probability, a measure of the likelihood that *v* will activate the target concept.
*E*(*v*)	The expected number of activations that *v* will provide if *v* activates the target concept.
*WE*(*v*)	The weighted expected gain of *v*, namely *E*(*v*) weighted by *ap*(*v*).

We wish to select locally influential nodes, that can spread the controllable concept and affect the spread of the target concept. This is similar to the notion of betweeness [10, 45], since selecting nodes with a high betweeness means that they are likely to be encountered by the target concept and to reach a higher number of nodes. Determining betweeness is computationally expensive, since it requires the calculation of a large number of shortest paths. Therefore, we need an alternative method to estimate the influence of a node. Nodes that are likely to both activate the target concept and provide a high number of expected activations are likely to be locally influential, therefore MPG aims to select such nodes.

**Algorithm 1**: Greedy Algorithm using MPG Heuristic

**input**: Set of nodes in the network, *V*; Set of nodes that will attempt to spread concept *t* in the next time step, *S*_*t*_; probability threshold, *θ*; Number of seeds to select, *s*

**output**: Seed set for controllable concept *c*, *S*_*c*_

**for** *v* ∈ *V* **do**

 *#Initialise the activation probability (ap) and expected gain (E) for node v*.

 *ap*(*v*) ← 0;

 *E*(*v*) ← 0;

**end**

**for** *n* ∈ *S*_*t*_ **do**

 **foreach** {*v*|*IR*_*n*,*v*_ > *θ*} **do**

  *#Each node reachable by n has some chance of activating the concept t from n, and so we update their activation probability accordingly*.

  *ap*(*v*) ← *ap*(*v*) + *IR*_*n*,*v*_;

 **end**

**end**

#Only explore nodes with some chance of activating the target concept

**foreach** {*v*|*ap*(*v*) > 0} **do**

 $E(v)=\sum_{u|IR_{n,v}>\theta }IR_{n,v};$

 *#We weight expected gain by the chance of activating the target concept*

 *WE*(*v*) ← *E*(*v*) × *ap*(*v*);

**end**

**while** |*S*_*c*_| < *s* **do**

 *#Select node n with highest weighted expected gain and add it to the seed set*

 *v* ← {*n*|∀_*x*∈*V*_ *WE*(*n*) > *WE*(*x*)};

 *S*_*c*_ ← *S*_*c*_ ∪ *v*;

 *#v’s adopting context will have changed, and so we must update the expected gain of nodes that included* *v in its calculation*.

 **foreach** {*u*|*IR*_*n*,*v*_ > *θ*} **do**

  *Recalculate* *E*(*u*) *taking new concept interaction with c into account*;

  *WE*(*u*) ← *E*(*u*) × *ap*(*u*);

 **end**

 *#v’s spreading context will have changed, and so any node that receives influence from a node in* *S*_*t*_ *through v will need to have its activation probability*, *ap*(*u*), *updated*.

 **foreach** {*u*|*v* ∈ *MIP*(*w*, *u*) ∧ *w* ∈ *S*_*t*_} **do**

 *Recalculate* *ap*(*u*) *taking new concept interaction with c into account*;

 *WE*(*u*) ← *E*(*u*) × *ap*(*u*);

 **end**

**end**

**return** *S*_*c*_;

We assume that MPG has full knowledge of the network, including which nodes have the target concept active. A similar assumption was made by the authors of MoBoo [37], as some level of knowledge of the target concept is needed to be able to indirectly manipulate it. While not always realistic, there are cases where we may know that an individual has a concept active and be unable to directly interact with that concept. For instance, consider a disease such as bird flu. When it is first introduced into a population, there is no vaccine, but information about the disease and its symptoms can help to limit its spread through a population. Alternatively, we can often learn of someone’s political affiliation, but it is difficult to directly encourage others to adopt that affiliation. Instead, a person may be influenced to read news stories that cause a change in opinion. In both cases we can see how, while we may not be able to control the target concept, we may be able to learn which individuals have it active and so manipulate its spread.

### 5.1 Activation probability

If an edge exists in a network from node *v* to node *u*, then *v* can exert influence on *u* in relation to a concept *c*. This influence, *I*_*v*,*u*_(*c*), is used in an influence spread model to determine whether *u* will activate concept *c* based on *v*’s influence. We define the set *S*_*t*_ as the set of nodes that are able to exert influence with regard to concept *t* in the next time step. For the ICM, this is the set of nodes that activated *t* in the previous time step or, if we are at the start of a cascade, the initial seed set. For the LTM, this is the set of nodes that currently have the target concept active, since nodes in the LTM always exert influence, to model the gradual effect of peer pressure.

For each node *v* ∈ *S*_*t*_, the influence reaching node *u* from *v*, for concept *t*, is dependant on the most influential path from *v* to *u*, *MIP*_*t*_(*v*, *u*). For a given path *P* = {*v* = *v*_1_ → *v*_2_ → … → *v*_*n*−1_ → *v*_*n*_ = *u*}, the strength of the influence reaching *u* from *v* is defined as:

$$I_{P}=\prod_{v_{i},v_{i+1}\in P}CI_{v_{i},v_{i+1}}\left(t\right)$$ (4)

If a node in the path has activated the controllable concept, the influence it receives and exerts in relation to the target concept will be modified according to the concept relationship between the controllable and target concept. The use of *CI*_*v*,*u*_(*t*) in the definition of *I*_*P*_ allows for that relationship to be accounted for. *MIP*(*v*, *u*) can then be defined as:

$$MIP\left(v,u\right)=argmax_{P\in AP(v,u)}\left(I_{P}\right)$$ (5)

where *AP*(*v*, *u*) is the set of all paths that start with *v* and end in *u*. This means the influence received by *u* from *v*, denoted as *IR*(*v*, *u*), is equal to *IR*(*v*, *u*) = *I*_*MIP*(*v*,*u*)_. If *v* cannot reach *u* then *MIP*(*v*, *u*) does not exist and *IR*(*v*, *u*) = 0. Calculating *IR*(*v*, *u*) for every node that can be reached by *v* is computationally expensive. As such, for each node *v* ∈ *S*_*t*_, only the *IR*(*v*, *u*) values for the local area are calculated. Starting at *v*, we explore the local neighbourhood recursively, calculating the most influential path for each new node encountered until the *IR*(*v*, *u*) values drop below a chosen threshold, *θ*. Since the influence received from *v* decreases as path length increases, eventually this threshold will be reached and exploration will stop. We can account for this in an expanded definition of *IR*(*v*, *u*):

$$IR\left(v,u\right)=\{\begin{array}{cc} I_{MIP(v,u)} & \text{if}\;\exists MIP\left(v,u\right)\wedge I_{MIP(v,u)}>\theta \\ 0 & \text{otherwise} \end{array}$$ (6)

The activation probability of a node, *u*, can now be defined as the sum of the influence received from all nodes with the target concept active:

$$ap\left(u\right)=\sum_{v\in S_{t}}IR\left(v,u\right)$$ (7)

**Fig 2:**
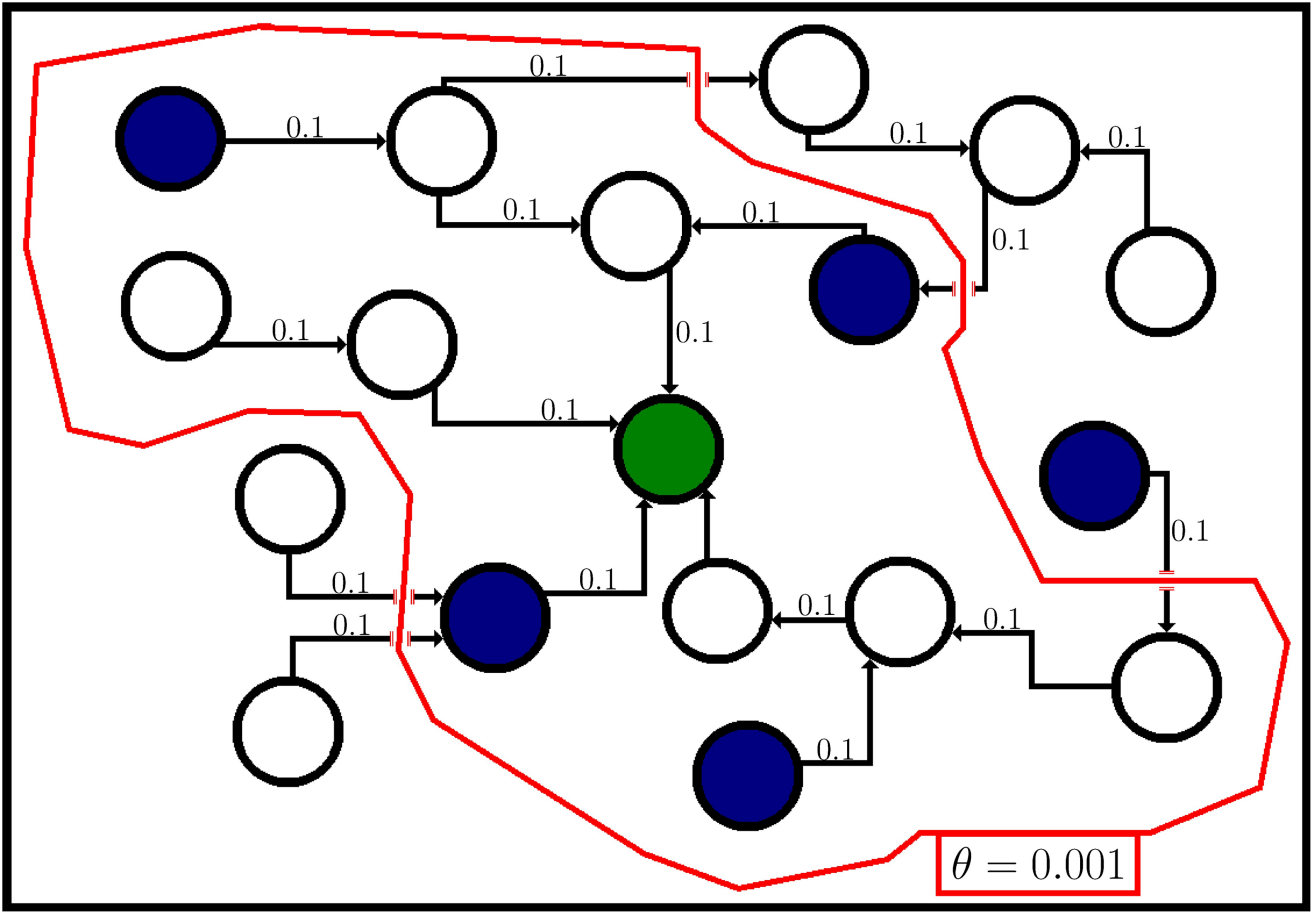
Illustration of how a node with the target concept active can contribute to another node’s activation probability in the ICM, where *p* = 0.1. Blue nodes have the target concept active, and the red border signifies a propagation probability of *θ* = 0.001. A concept spreading from a node within that border will reach the green node with a probability higher than *θ*, a concept spreading from outside the border will reach the green node with a probability lower than *θ* and so does not contribute to the green node’s *ap*(*green*).

Fig 2 illustrates the calculation of the activation probability of *u*, represented in the figure as the green node. The blue nodes have the target concept active, and those within the red area can send influence to *u* equal to or greater than *θ* and so contribute to *ap*(*u*). The nodes outside the exploration range, which send influence to *u* less than *θ*, do not contribute to *ap*(*u*). Note that in this case, the controllable concept has yet to be introduced, and so each edge has the same probability of propagating the target concept.

### 5.2 Expected gain

With the activation probability calculated, we now consider a node’s expected activations for the target concept. This is simply calculated as the sum of the influence that a node, *u*, exerts on all other nodes within the network:

$$E\left(u\right)=\sum_{w}IR\left(u,w\right)$$ (8)

**Fig 3:**
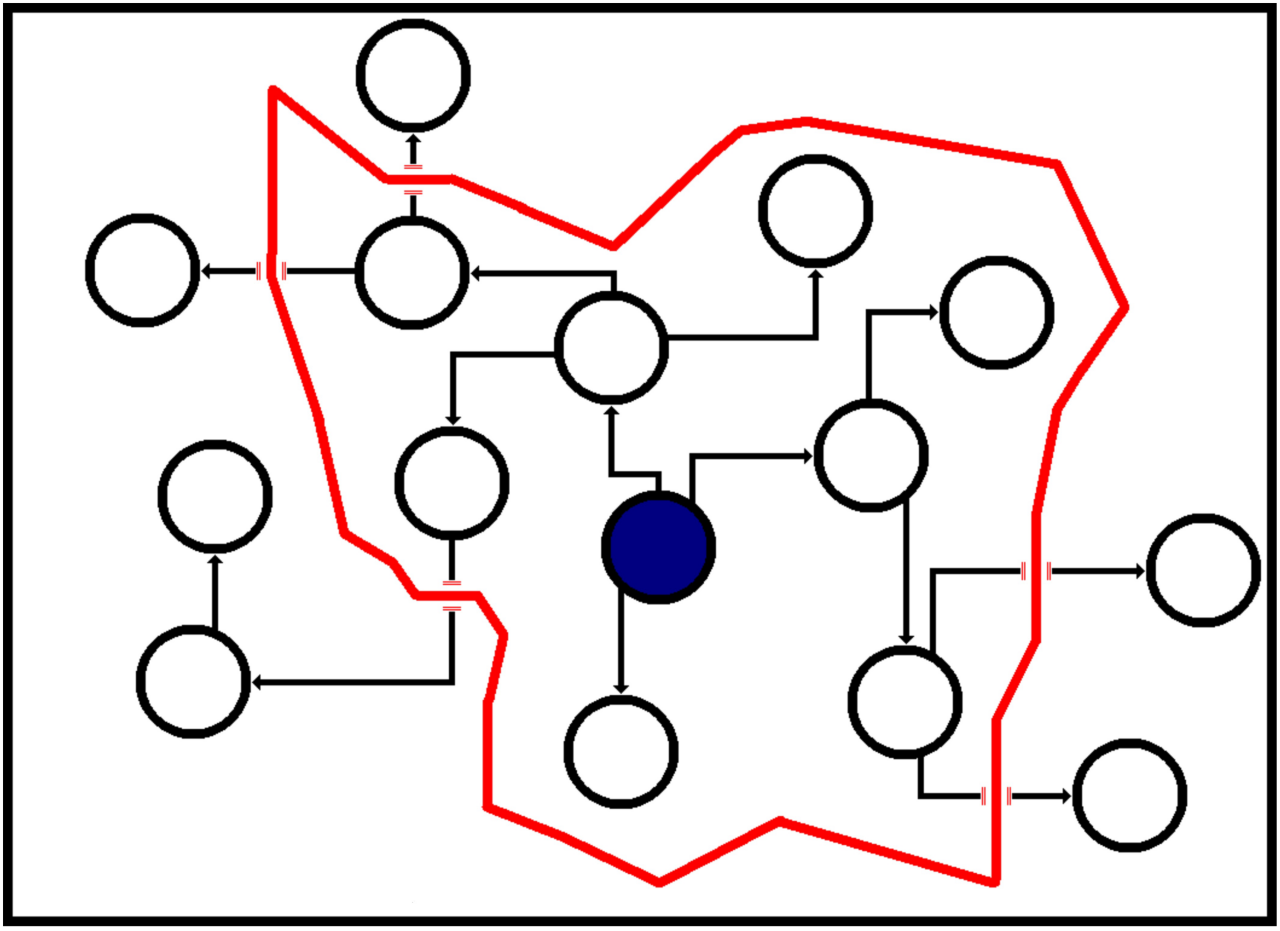
Illustration of reachability, where the red border represents a propagation probability of *θ*. Nodes inside the red area have a probability higher than *θ* to be reached by a concept spreading from the blue node and so contribute to its expected gain. Nodes outside the red area have a probability lower than *θ* to be reached and so do not contribute to the expected gain of the blue node.

Due to the definition of *IR*(*u*, *w*), *E*(*u*) can be calculated efficiently, as it will only need to the explore a small local area of the network. This is illustrated in Fig 3, where nodes outside of the red area receive an amount of influence less than *θ* and so do not contribute to *E*(*v*).

To identify influential nodes that are likely to be encountered, the expected gain of a node is weighted by the probability that it will activate the target concept. This weighted expected gain, *WE*(*u*), for a node *u* represents the expected value of *u* to the spread of the target concept, is defined as:

WE(u)=E(u)×ap(u) (9)

A high *WE*(*u*) value indicates a node is both likely to activate the target concept and provides a high expected gain for the target concept. As such, we select the node with the highest weighted expected gain, *WE*(*u*), as a seed node for the controllable concept and we update the value of *WE*(*v*) for any node *v* that will be affected by the presence of the controllable concept at node *u*. The selection process then repeats, selecting the node with the highest *WE*(*u*) value, and recalculating *WE*(*v*) for affected nodes until the seed set for the controllable concept reaches the desired size.

## 6 Experimental set-up

We evaluate the effectiveness of our proposed heuristic using the experimental parameters given in Table 3. For both the indirect influence maximisation and indirect influence limitation problems, we compare the performance of MPG against heuristics used to maximise the spread of the secondary concept and the MoBoo heuristic for indirect influence maximisation. As previously stated, the code used to run the simulations is obtainable from: https://github.com/JamesArchbold/ConceptInteraction.

**Table 3:** Experimental parameters.

Parameter	Values
Graph size (nodes)	1000, 5000, 10000, 25000, 50000, 100000, 250000
Clustering exponent for small-world networks	0.25, 0.75
Number of edges added each evolution step for scale-free networks	4, 8
Real-world network samples used	DBLP, CA-CondMat, soc-Epinions1
Seed set size	10, 25, 50, 100, 250, 500
*r* values	0, 0.2, 0.4, 0.6, 0.8, 1.2, 1.4, 1.6, 1.8, 2
Burn-in time steps	0, 2, 5

In this evaluation, for the ICM we assume *θ* = 0.001, meaning that a path must have an influence strength above 0.001 to be included in the calculation of *ap*(*v*) and *E*(*v*) values, limiting our considerations to a local area around each node with the target concept active. This setting is equivalent to SIR model where the probability of deactivation is 1, however the majority of influence spread studies use terms associated with the ICM, and so we use ICM terminology in this paper.

When evaluating MPG in the LTM, *θ* instead represents the length of a path. Due to the variance in influence strength present in the LTM, the amount of the network we explore can vary drastically. Since a typical cascade does the majority of its spreading in the first few time steps we only consider paths of length 4 or less, meaning that from each node we explore their 3-hop neighbourhood.

For the ICM, we assume that the probability of a concept spreading from an infector to a receiver is initially *p* = 0.1. This value is then modified by the interactions between the different concepts active on the infector and receiver. For the LTM, nodes have a threshold selected from a Gaussian distribution with a mean of 0.8 and standard deviation of 0.05. This means that the average node is difficult to activate, which mirrors the low chance of infection present in the ICM.

We acknowledge that both of these models are simplistic, and that the results we obtain are limited by the restrictions of the models. In this paper, we focus on demonstrating the feasibility of indirect concept manipulation and so we use two of the most widely used models in the study of influence spread. In the future, we intend to perform investigations with other, more dynamic models such as SIS and the SIR model with a wider range of deactivation probabilities.

We compare the performance of MPG against other heuristics for the indirect influence maximisation and the indirect influence limitation problems. In particular, we compare its performance against degree discount [4] and MoBoo [37]. These heuristics have been shown to perform well in related settings and so provide comparators for evaluating the performance of MPG. In addition, we will compare against the performance of simple heuristics, such as highest degree and random selection, to provide a baseline.

The target concept has a randomly selected seed set in all our experiments. We use the following heuristics to select a seed set for the controllable concept:

- Random—randomly chosen nodes are selected.
- Highest Degree—nodes with the highest degree are selected.
- Single Discount—nodes with the highest degree of nodes not already selected for the seed set are selected.
- Degree Discount—nodes with the highest 1-hop expected gain are selected.
- MoBoo—nodes with the most probable paths of activation are selected.
- MPG—nodes likely to activate target concept, with high expected gain, are selected.

Note that we do not use the highest infectees heuristics proposed by Budak *et al*. [39], as simulating cascades is often an expensive approach to estimating the effectiveness of seed sets. Furthermore, the heuristic assumes blocking concepts, unlike MoBoo, which can be interpreted as activating a concept that does not spread on the nodes it selects.

The primary target concept will spread for a given number of time steps, referred to as the burn-in time, before we introduce and select seeds for the controllable concept. We evaluate the heuristics using a variety of burn-in times in order to explore the impact of burn-in time on the effectiveness of the various heuristics. We focus on short burn-in times, preventing the controllable concept being introduced after the target concept has stopped spreading and at which point we would be unable to manipulate it.

**Fig 4:**
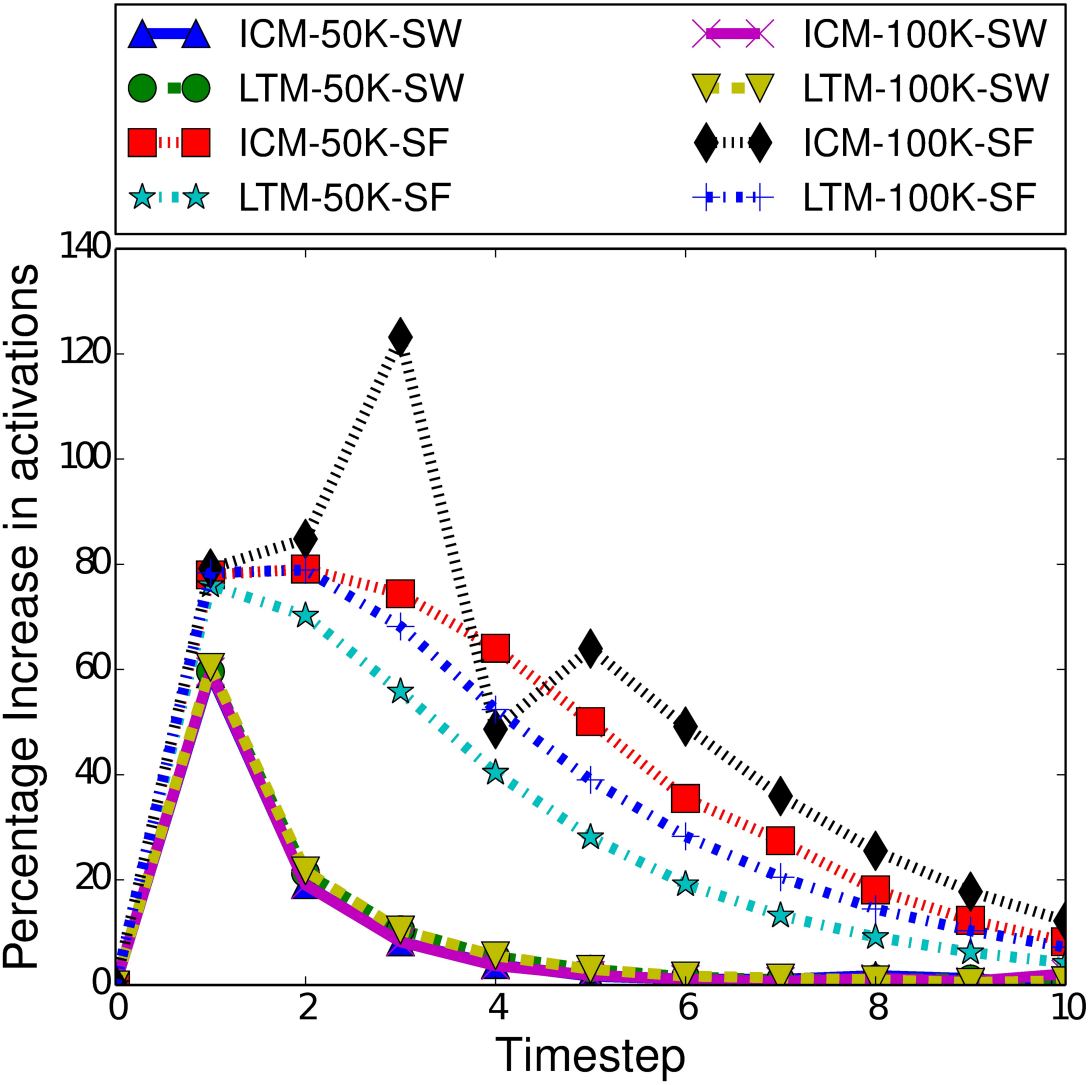
Mean percentage increase of activations in the first 10 time steps of a cascade, for a single concept using a randomly selected seed set of 250 nodes.

We performed a small selection of initial cascades, where there is one concept spreading from a randomly selected seed set of 250 nodes, using either the ICM or LTM as described above. These tests used both scale-free and small-world networks of 50000 or 100000 nodes. As shown in Fig 4, we see an increase in the number of infections each round in the early time steps, but always by time step 4, the number of new infections begins to rapidly decline. Note this happens for both influence spread models, demonstrating why we wish to intervene early and so focus on early burn-in times. Furthermore, we test a range of boosting and inhibiting relationship strengths. This allows us to determine whether different strategies may be more viable at different levels of relationship strength.

Our evaluation of these heuristics requires a variety of networks. Real-world networks can be characterised by a number of topological features [46, 47]. Two characteristics that are often considered are the small-world and scale-free properties, both of which are often present in real-world networks [48, 49]. As such, synthetic small-world and scale-free networks are both used to evaluate the performance of the heuristics, with a range of sizes as detailed in Table 3. The results from these networks can help to inform how MPG may perform in real-world situations.

The small-world networks used in this paper have a clustering exponent of either 0.25 or 0.75. These are generated using the Kleinberg small-world generator provided in the JUNG graph framework (http://jung.sourceforge.net/) [50]. The scale-free networks are constructed through the use of the Barabási-Albert generator provided in JUNG [51]. This generator begins with an initial set of unconnected nodes and introduces a new node at each evolution step. This node then gains a number of edges, with connections chosen randomly through preferential attachment. We begin with 10 unconnected nodes and add either 4 or 8 edges in each step. For each combination of network size, type and network characteristics, we generate 100 networks.

We also run tests on selected real-world networks, taken from the Stanford SNAP project networks (http://snap.stanford.edu/data), namely *DBLP*, *CA-CondMat* and *soc-Epinions1*. Real-world networks are not strictly small-world or scale-free and instead can display properties of both types of network [52, 53]. Details of the characteristics of the real-world network samples we utilise are provided in Table 4.

**Table 4:** Characteristics of the real-world network samples used for evaluation.

Network	Nodes	Edges	Avg. Degree	Avg. Clustering Coefficient	Num. of Triangles	Diameter
DBLP	317080	1049866	3.31	0.6324	2224385	21
CA-CondMat	23133	93497	4.04	0.6334	173361	14
soc-Epinions1	75879	508837	6.7	0.1378	1624481	14

For networks of 10000 nodes or less, we perform simulations for each combination of *r* value, burn-in time, controllable concept heuristic and seed set size under 100. For networks of 25000 nodes or more, we use each seed set size of 100 or above. This means that for each controllable concept heuristic, we have 100 results for each combination of *r* value, seed set size, burn-in time, network type and network size. For the real-world networks we run 100 simulations for each combination of parameters, using seed set sizes of 100 or above. We also performed two tailed t-tests to compare the performances of the various heuristics. For each environment, we performed the test on each possible pairing of heuristics.

With these experiments we aim to evaluate the feasibility of indirect influence manipulation in a social network. The main limitation of this work is that while we use synthetic networks and models that have been widely used, we focus on simulation rather than real-world validation. Ideally, after an initial period of testing, a real-world study would be performed. As discussed in Section 2.4, the few real-world studies that exist are small in scope, and focus on the problem of uncertainty. Instead, we would wish to use a large network for which topological information is available, such as an online social network. We would then select individuals to generate content that relates to another, trending, topic but without directly linking to it. From there we would track the individual’s neighbours that begin to spread information on the trending topic. However, such a study is difficult to perform as many social networks are, understandably, protective of their data. Furthermore, there is an issue of how to incentivise the individuals we choose to generate content. For now, we focus on empirically validating a method of indirect influence manipulation, since real-world studies are expensive to perform, are time consuming, and potentially raise privacy concerns. Our view is that demonstrating the effectiveness of a heuristic through simulation is an important first step that should precede any real-world application.

## 7 Results for indirect concept maximisation

We evaluate the performance of MPG for both the ICM and LTM for the indirect influence maximisation problem, across three types of network namely small-world, scale-free and real-world. In this problem, the controllable concept has a boosting relationship with the target concept and we aim to maximise the spread of the target concept.

### 7.1 Small-world networks

We begin our assessment of the performance of the different heuristics with small-world networks. We first consider the ICM and then the LTM, discussing the impact of the different parameters explored in this study.

#### 7.1.1 The Independent Cascade Model

When considering small-world networks in the ICM, we see that MPG performs well when the burn-in time is 0, consistently outperforming all other heuristics in increasing the number of target concept activations, as shown in Table 5. MoBoo is the only other heuristic that performs close to MPG, with the other degree-based heuristics all performing poorly. This is to be expected, due to MoBoo being the only other heuristic that accounts for possible concept interactions and was explicitly designed for concept spread maximisation. Table 5 summarises the performance of the heuristics when the concept relationship is strong. We note that, when comparing various graph types, the size of the graph and its clustering exponent have no significant effect on the performance of any heuristic. The size of the seed set does affect performance, as is to be expected. The more initial seed nodes there are, the further we expect concepts to spread. Importantly, the rank order of the heuristics’ performance does not change, but each heuristic increases the number of activations by a similar magnitude.

**Table 5:** Average infections for the target concept for small-world networks in the ICM, with no burn-in time and a r value of 2, with standard deviation in brackets, and the best performing heuristic in bold.

Network Size	Clustering Exponent	Seed Set Size	MPG	MoBoo	Degree Discount	Single Discount	Degree
25K	0.25	100 Seeds	**292.61 (31.66)**	282.61 (30.64)	232.55 (22.04)	233.04 (22.1)	232.9 (22.4)
25K	0.25	250 Seeds	**753.31 (45.39)**	708.28 (43.8)	608.07 (46.03)	609.56 (48.75)	611.95 (47.49)
25K	0.25	500 Seeds	**1546.92 (76.15)**	1435.6 (66.19)	1313.7 (76.7)	1316.67 (80.12)	1318.14 (77.7)
25K	0.75	100 Seeds	**294.4 (33.53)**	281.99 (28.62)	232.31 (23.02)	231.97 (22.98)	233.35 (23.39)
25K	0.75	250 Seeds	**750.56 (54.53)**	714.73 (45.28)	609.84 (43.65)	608.37 (41.87)	610.89 (41.92)
25K	0.75	500 Seeds	**1540.68 (72.81)**	1438.09 (68.81)	1304.88 (70.24)	1308.79 (68.78)	1325.89 (70.5)
50K	0.25	100 Seeds	**295.04 (30.99)**	281.87 (29.64)	227.02 (21.95)	226.89 (21.73)	227 (22.39)
50K	0.25	250 Seeds	**733.58 (53.79)**	704.06 (46.73)	584.67 (39.54)	585.59 (40.49)	586.34 (41.43)
50K	0.25	500 Seeds	**1499.54 (73.24)**	1422.68 (69.56)	1216.91 (65.04)	1216.42 (67.51)	1224.42 (64.51)
50K	0.75	100 Seeds	**291.01 (28.6)**	281.26 (32.48)	228.53 (21.18)	228.53 (21.19)	228.19 (20.97)
50K	0.75	250 Seeds	**750.06 (51.49)**	705.2 (46.78)	582 (38.26)	583.05 (38.93)	583.35 (37.4)
50K	0.75	500 Seeds	**1504.87 (69.69)**	1426.12 (66.18)	1211.13 (63.93)	1215.25 (61.03)	1216.53 (61.24)
100K	0.25	100 Seeds	**294.17 (30.48)**	275.92 (29.11)	224.65 (21.2)	224.64 (21.2)	224.46 (21.37)
100K	0.25	250 Seeds	**731.65** (48.26)	703.81 (46.22)	571.7 (38.64)	571.72 (38.31)	571.09 (36.94)
100K	0.25	500 Seeds	**1475.36 (73.13)**	1408.22 (62.86)	1164.93 (58.47)	1163.93 (58.31)	1166.44 (54.58)
100K	0.75	100 Seeds	**292.47 (29.34)**	280.58 (28.36)	225.19 (20.52)	225.18 (20.53)	225.45 (20.56)
100K	0.75	250 Seeds	**731.28 (52.45)**	701.58 (44.84)	573.45 (38.25)	573.16 (38.63)	574.07 (37.54)
100K	0.75	500 Seeds	**1476.99 (79.42)**	1416.78 (67.01)	1163.53 (55.28)	1164.8 (53.69)	1168.76 (52.92)
250K	0.25	100 Seeds	**289.04 (30.34)**	279.91 (28.44)	223.95 (20.39)	223.95 (20.39)	223.75 (20.09)
250K	0.25	250 Seeds	**730.09 (43.66)**	697.2 (39.09)	561.74 (34.8)	561.74 (34.8)	562.71 (35.57)
250K	0.25	500 Seeds	**1462.48 (71.56)**	1407.13 (62.7)	1131.7 (51.8)	1132.02 (50.44)	1130.32 (50.02)
250K	0.75	100 Seeds	**288.33 (30.81)**	279.53 (28.95)	224.46 (20.71)	224.46 (20.72)	224.62 (20.77)
250K	0.75	250 Seeds	**731.19 (51.08)**	704.46 (43.99)	560.76 (35.36)	561.12 (35.34)	561.89 (36.19)
250K	0.75	500 Seeds	**1467.19 (69.34)**	1400.71 (65.79)	1135.2 (53.17)	1135.48 (53.09)	1135.37 (52.41)

**Fig 5:**
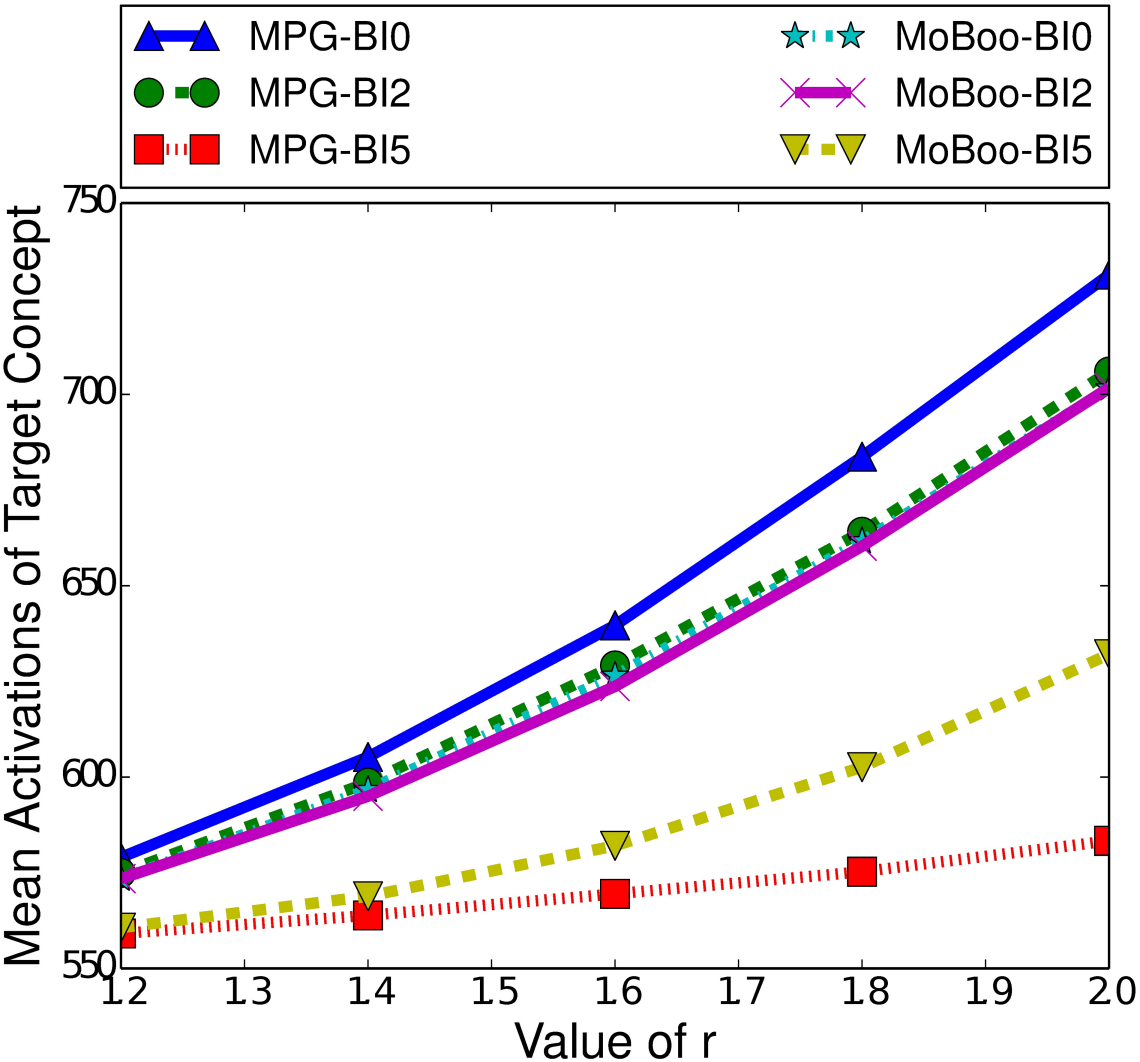
Mean activations of the target concept given the heuristic used to select the boosting concept in the ICM, for small-world networks of 100000 nodes, with a clustering coefficient of 0.75, a seed set of 250 nodes and a variety of burn-in (BI) times.

Two factors that do notably affect the performance of MPG and MoBoo are the length of the burn-in time and the strength of the relationship between the target and controllable concept. Fig 5 highlights the difference in performance between MPG and MoBoo for a variety of burn-in times and relationship strengths. As the relationship strength increases, we see both heuristics improve their performance, as a stronger relationship will naturally make MPG and MoBoo more effective, as they aim to maximise the number of interactions between the concepts. With a strong concept relationship, any interaction between the target and controllable concept greatly increases the probability of the target concept spreading, which can in turn facilitate further interactions.

Fig 5 also demonstrates the effect of the burn-in time on the performance of the heuristics. Both heuristics demonstrate decreased performance with higher burn-in times, as is expected based on Fig 4. A typical cascade gains most of its activations in the first few time steps, and a higher burn-in time means that there are fewer activations occurring and so less chance of interactions taking place. With no interactions, we cannot indirectly affect the spread of a concept. We also note that MPG is more affected by burn-in time than MoBoo. When the burn-in time is 0, we see a significant difference between the two heuristics (*p* < 0.05) in favour of MPG. However, at a burn-in time of 2, there is minimal difference between the two heuristics, and with a burn-in of 5 MoBoo begins to outperform MPG. At weaker relationship strengths, we can see that the difference is minimal and not statistically significant (*p* > 0.2). However, once the r value exceeds 1.4, MoBoo significantly outperforms MPG when the burn-in time is 5 (*p* < 0.05).

While both heuristics calculate the gain of selecting a given node, the method utilised by MoBoo seems to mitigate the impact of fewer activations occurring in later time steps.

#### 7.1.2 The Linear Threshold Model

When concepts spread using the LTM, we see a similar performance in small-world graphs as we do for when concepts use the ICM. As Table 6 highlights, MPG continues to out-perform the other heuristics when there is no burn-in time in small-world networks. A major difference is the magnitude of activations. When comparing similar network and seed set sizes, we see a larger number of activations for all heuristics in the LTM when compared to the ICM. Nodes in the LTM continuously exert influence once they activate a concept and so, unlike in the ICM, a node that activates the controllable concept after activating the target concept can still be affected. This means there will be more concept interactions, leading to the boosting relationship being more effective. Note that this is true for small-world networks due to the low variability of node degree, meaning that most nodes will have a degree close to the average. In the LTM, nodes typically require multiple neighbours to be active before they activate a concept and so these additional interactions contribute effectively to the number of activations. In networks with a higher number of low degree nodes, we expect to see fewer activations in the LTM. We also see that MoBoo has its relative performance affected. In the ICM we observed that MoBoo performed similarly to MPG, but in the LTM there is a much larger difference between the two.

**Table 6:** Average infections for the target concept for small-world networks in the LTM, with no burn-in time and a r value of 2, with standard deviation in brackets, and the best performing heuristic in bold.

Network Size	Clustering Exponent	Seed Set Size	MPG	MoBoo	Degree Discount	Single Discount	Degree
50K	0.25	250 Seeds	**1936.24 (99.61)**	1075.22 (88.61)	765 (66.88)	766.23 (68.52)	767.82 (67.93)
50K	0.25	500 Seeds	**3676.38 (125.71)**	2148.98 (124.69)	1787.41 (136.84)	1780.76 (136.39)	1789.14 (137.51)
50K	0.75	250 Seeds	**1912.98 (94.7)**	1084.7 (99.27)	763.74 (69.95)	761.71 (70.13)	764.57 (70.06)
50K	0.75	500 Seeds	**3648.86 (125.43)**	2170.27 (139.77)	1788.25 (138.46)	1784.9 (128.43)	1791.25 (135.14)
100K	0.25	250 Seeds	**1953.78 (105.17)**	1065.89 (82.71)	696.63 (61.35)	696.43 (61.53)	697.32 (61.8)
100K	0.25	500 Seeds	**3826.88 (130.51)**	2175.4 (119.22)	1519.09 (97.44)	1518.35 (98.02)	1519.8 (102.06)
100K	0.75	250 Seeds	**1950.12 (103.37)**	1085.99 (88.14)	678.0 (58.47)	678.16 (58.87)	678.07 (58.56)
100K	0.75	500 Seeds	**3830.86 (130.47)**	2181.32 (139.24)	1502.97 (105.5)	1499.34 (104.39)	1505.76 (103.92)
250K	0.25	250 Seeds	**1976.35 (96.6)**	1022.19 (91.59)	638.45 (43.97)	638.63 (44.11)	638.75 (44.14)
250K	0.25	500 Seeds	**3923.19 (158.47)**	2136.08 (122.86)	1341.01 (75.75)	1340.95 (75.39)	1341.8 (76.43)
250K	0.75	250 Seeds	**1998.11 (116.46)**	1032.33 (81.96)	643.47 (45.07)	643.37 (45.08)	643.82 (44.89)
250K	0.75	500 Seeds	**3944.85 (149.46)**	2143.61 (137.61)	1357.81 (87.10)	1357.87 (85.63)	1358.46 (86.93)

**Fig 6:**
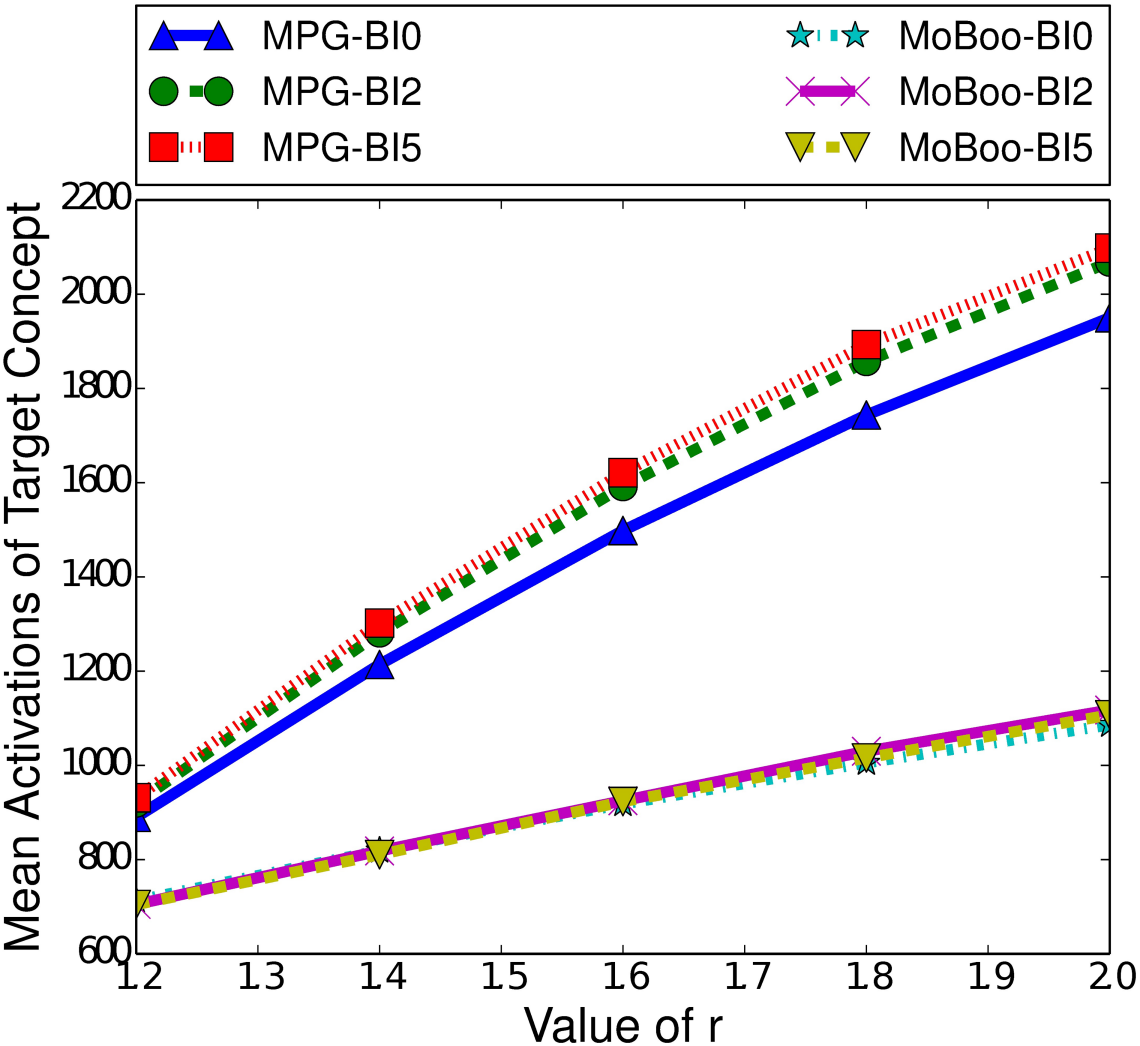
Mean activations of the target concept given the heuristic used to select the boosting concept in the LTM, for small-world networks of 100000 nodes, with a clustering coefficient of 0.75, a seed set of 250 nodes and a variety of burn-in (BI) times.

Another significant difference between the ICM and the LTM is the effect of burn-in time. As Fig 6 demonstrates, a longer burn-in time actually slightly improves the performance of MPG. If we compare the difference in performance between a burn-in time of 0 and 2, we see a noticeable increase in the performance of MPG. Furthermore, we see a diminished increase between a burn-in time of 2 and 5. At higher burn-in times, in the LTM, there will be more actively spreading nodes, unlike in the ICM where there are less. For example, consider introducing the controllable concept at time step 0, with a seed set size of 100 for both concepts. This means that there are 100 nodes that can spread the target concept when the controllable concept is introduced. If instead, we introduce the controllable concept at time step 2, the target concept will have spread and there may be 200 nodes actively spreading the target concept. In the LTM, once a node activates a concept, it as always capable of spreading that concept and so introducing the controllable concept at time step 2 in this scenario is equivalent to introducing it at time step 0 but with a seed set size of 200 for the target concept. We have previously seen that a higher number of nodes with the target concept active increases the performance of MPG, and this remains true here.

### 7.2 Scale-free networks

Scale-free networks differ to small-world networks in the way that nodes are connected to each other. In scale-free networks the degree distribution follows a power law, meaning there are a small number of nodes with a very high degree while the majority of nodes will have a very low degree. In this section we explore how this characteristic affects indirect influence maximisation, across both the ICM and LTM.

#### 7.2.1 The Independent Cascade Model

In scale-free networks, for the ICM there is little difference between the degree-based heuristics and MPG in terms of performance, as shown in Table 7, although MPG does consistently outperform MoBoo. Across the different network types and sizes, the degree-based heuristics consistently perform best, followed by MPG and then MoBoo. The power law degree distribution present in scale-free networks clearly lessens the advantage of the exploration performed by MPG and MoBoo.

**Table 7:** Average infections for the target concept for scale-free networks in the ICM with no burn-in time, and a r value of 2, with standard deviation in brackets, and the best performing heuristic in bold.

Network Size	Edges Added	Seed Set Size	MPG	MoBoo	Degree Discount	Single Discount	Degree
25K	4	250	12220.48 (183.12)	11580.56 (294.18)	**12446.73 (159.01)**	12445.14 (147.85)	12438.59 (143.63)
25K	8	250	22414.8 (71.87)	22197.01 (136.53)	22465.05 (75.49)	**22473.18 (70.13)**	22463.33 (71.41)
25K	4	500	13103.45 (139.27)	12302.31 (229.83)	**13327.3 (139.94)**	13301.18 (145.44)	13283.67 (130.45)
25K	8	500	22514.37 (66.69)	22275.77 (99.93)	**22552.31 (64.63)**	22549.23 (70.51)	22543.82 (54.05)
50K	4	250	23091.52 (311.76)	22226.45 (494.35)	**23672.06 (262.7)**	23627.58 (284.6)	23626.2 (265.27)
50K	8	250	44698.08 (112.16)	44294.13 (315.78)	44851.45 (118.24)	**44853.94 (128.74)**	44853.86 (119.93)
50K	4	500	24462.98 (233.44)	23181.27 (375.14)	**24877.7 (227.933)**	24838.64 (225.34)	24843.79 (221.45)
50K	8	500	44838.58 (100.48)	44382.93 (238.25)	44912.35 (103.95)	**44914.69 (97.97)**	44910.38 (105.17)
100K	4	250	44125.24 (505.32)	42936.25 (947.56)	45521.67 (451.93)	45544.39 (480.18)	**45563 (470.76)**
100K	8	250	89128.57 (193.06)	88556.89 (591.51)	89615.97 (220.06)	89614.2 (210.21)	**89621.14 (219.22)**
100K	4	500	46175.44 (422.25)	44318.91 (790.57)	**47281.32 (383.46)**	47253.77 (389.96)	47226.18 (397.04)
100K	8	500	89366.37 (156.95)	88574.56 (538.58)	89668.29 (178.1)	**89671.12 (175.29)**	89664.81 (165.27)

Unlike in small-world networks, we see that the seed set size does not significantly impact the performance of the different heuristics. This suggests that increasing the seed set size does not increase the area of the network that the seed set can reach, resulting in selecting new seeds whose reach largely overlaps with previously selected seeds. However, both network size and the number of edges added per node during creation affect performance. We can see that the performance of all heuristics is increased by approximately 85% when the number of edges is doubled, and similarly when the size of the network is doubled. Due to how the scale-free networks are constructed, increasing either of these parameters will result in the hub nodes of the network having higher degrees, increasing the chance of concept interaction, resulting in more activations.

**Fig 7:**
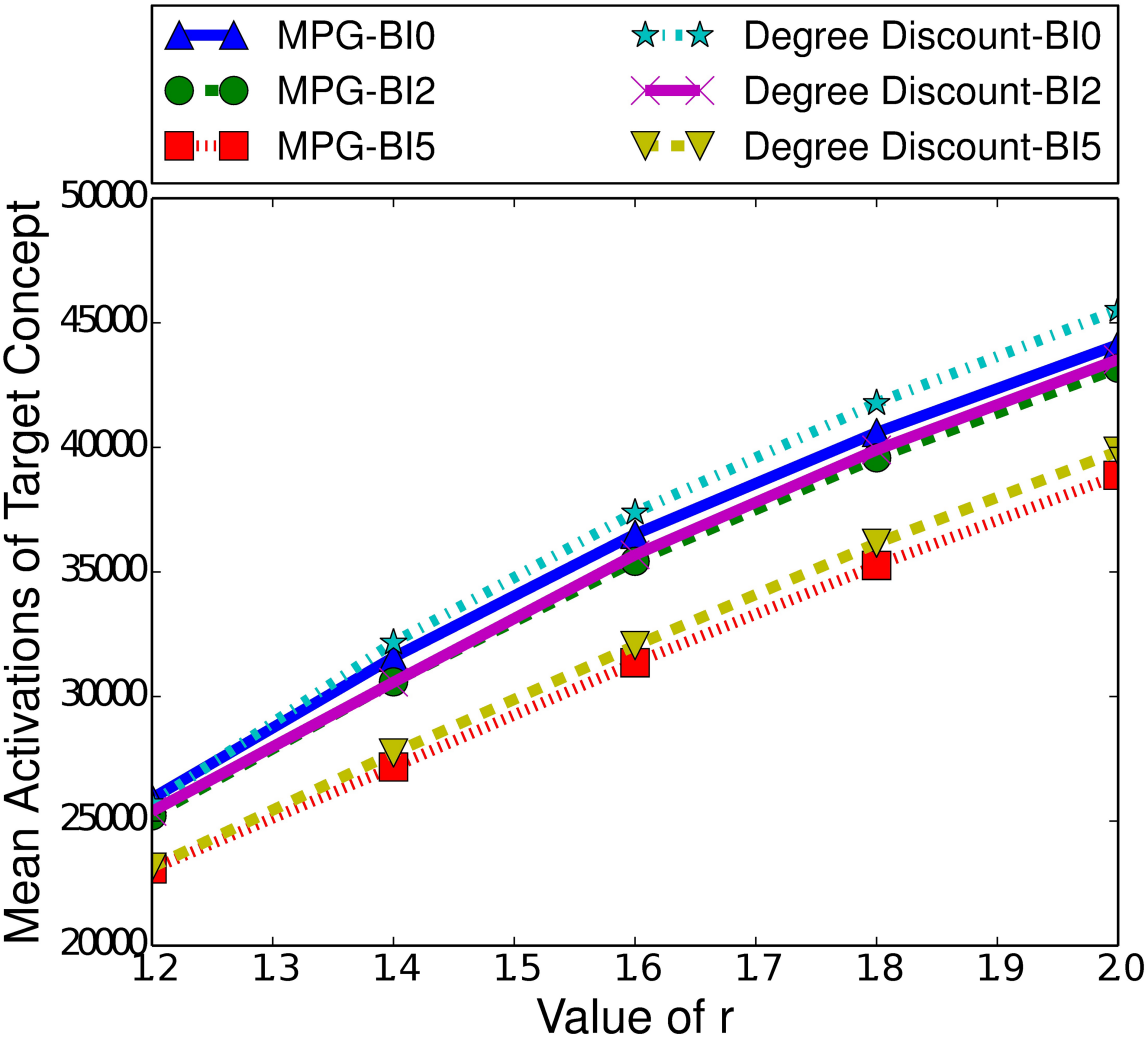
Mean activations of the target concept given the heuristic used to select the boosting concept in the ICM, for scale-free networks of 100000 nodes, with 4 edges added each round, a seed set of 250 nodes and a variety of burn-in (BI) times.

If we observe the performance of MPG and degree discount across different burn-in times and relationship strengths, as illustrated in Fig 7, we see that the burn-in time affects performance as is the case in small-world networks, although the impact is not as pronounced in scale-free networks. Furthermore, we see that degree discount increases its performance as the relationship strength increases. For both the ICM and LTM in small-world networks, the degree-based heuristics did not noticeably increase their performance as the relationship strength increased.

#### 7.2.2 The Linear Threshold Model

For the LTM in scale-free networks, we observe very similar results as for the ICM. As seen in Table 8, the degree-based heuristics all perform at a similar level and perform best, followed by MPG and then MoBoo. There are also fewer activations across all environments in the LTM than in the ICM. The power law degree distribution of scale-free networks means that the majority of nodes have a low degree, which can make the activation of a concept difficult in the LTM.

**Table 8:** Average infections for the target concept for scale-free networks in the LTM with no burn-in time, and a r value of 2, with standard deviation in brackets, and the best performing heuristic in bold.

Network Size	Edges Added	Seed Set Size	MPG	MoBoo	Degree Discount	Single Discount	Degree
25K	4	250	8417.3 (211.84)	7999.22 (288.65)	**9413.97 (135.01)**	9399.2 (146.04)	9400.17 (140.55)
25K	8	250	14305.99 (127.44)	14140.05 (128.87)	**14619.92 (113.19)**	14600.66 (116.21)	14598.96 (116.62)
25K	4	500	9641.65 (157.31)	8789.86 (220.2)	**10511.31 (143.2)**	10507.35 (127.35)	10501.49 (131.87)
25K	8	500	14884.06 (114.59)	14403.26 (158.81)	**15138 (105.56)**	15120.81 (101.55)	15120.9 (101.44)
50K	4	250	15157.62 (392.51)	14799.26 (400.04)	17140.48 (257.86)	17133.07 (240.9)	**17146.82 (243.42)**
50K	8	250	27806.82 (194.19)	27757.21 (291.11)	**28487.18 (167.36)**	28467.92 (178.31)	28465.26 (181.82)
50K	4	500	16934.76 (271.51)	15775.43 (390.22)	**18842.06 (187.93)**	18836.22 (180.88)	18837.58 (179)
50K	8	500	28655.92 (180.62)	28028.88 (257.51)	**29240.84 (166.86)**	29210.98 (170.26)	29208.1 (171.99)
100K	4	250	27842.81 (624.51)	27892.26 (654.94)	**31568.87 (395.58)**	31528.06 (414.58)	31536.62 (412.36)
100K	8	250	54513.87 (301.75)	54797 (2280.22)	**55931.31 (240.67)**	55902.32 (252.54)	55903.81 (252.19)
100K	4	500	30357.18 (532.83)	29205.02 (716.3)	**34220.32 (317.49)**	34203.87 (317.49)	34202.41 (333.25)
100K	8	500	55625.24 (264.51)	55223.84 (2896)	**57000.24 (265.43)**	56937.01 (267.99)	56948.37 (268.27)

**Fig 8:**
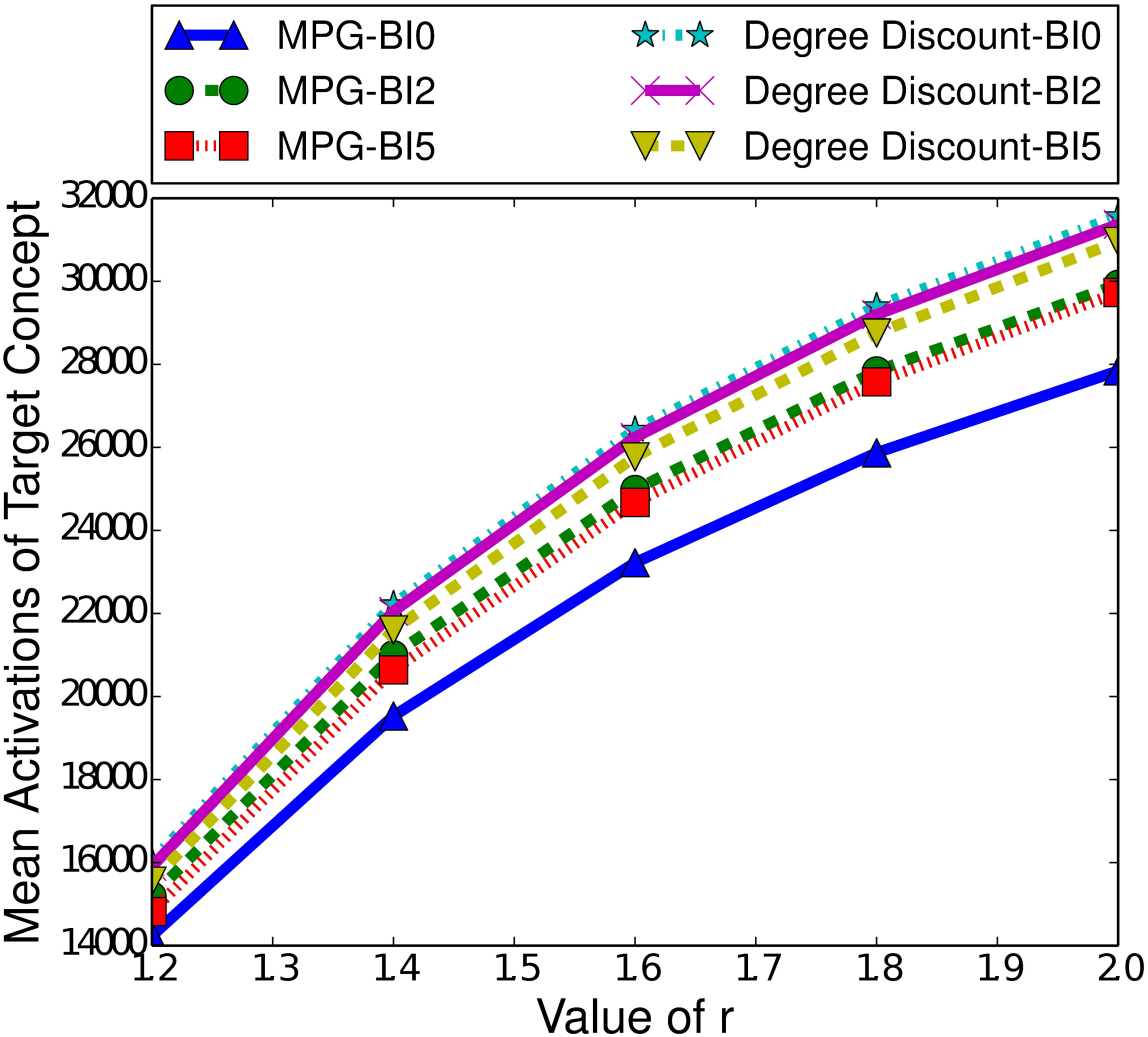
Mean activations of the target concept given the heuristic used to select the boosting concept in the LTM, for scale-free networks of 100000 nodes, with 4 edges added each round, a seed set of 250 nodes and a variety of burn-in (BI) times.

As expected, based on the results of the LTM in small-world networks, we see MPG increases its performance when the burn-in time is non-zero. As Fig 8 shows, while MPG does increase its performance, it never outperforms degree discount. We note that, unlike MPG, the performance of degree discount decreases as the burn-in time increases.

### 7.3 Real-world networks

Performing influence spread in the real-world is extremely difficult, due to the issues of defining what constitutes an individual being influenced, and of mapping the network. Therefore, we utilise simulations based on samples of real-world networks as discussed in Section 6.

#### 7.3.1 The Independent Cascade Model

In real-world topologies, for the ICM we observe a similar pattern of performance as seen in the synthetic scale-free networks. Degree-based heuristics perform the best, with no statistically significant difference between them, followed by MPG, and then MoBoo being outperformed by all other heuristics. From Table 9, we can see that both MPG and MoBoo are typically outperformed by the degree-based heuristics, despite their focus on utilising concept relationships. The difference in performance between MPG and the degree-based heuristics is smaller in both the CA-CondMat and the soc-Epinions1 networks. Despite DBLP having over 4 times the number of nodes as soc-Epinions1, it only has slightly more than twice the edges. Comparing DBLP to CA-Condmat we see that DBLP has nearly 14 times the number of nodes, and approximately 11 times the number of edges. These factors suggest that DBLP is a sparser network than soc-Epinions1 or CA-CondMat. MPG targets nodes close to the currently active nodes, which are likely to have a low degree in DBLP, decreasing the impact of the boosting relationship. In this case, attempting to maximise the spread of the controllable concept performs better, as we increase the chances of the target concept activating nodes in a path that leads to the few high degree nodes of DBLP.

**Table 9:** Average infections for the target concept for real-world networks in the ICM and a r value of 2, with standard deviation in brackets, and the best performing heuristic in bold.

Network	Seed Set Size	Burn-in Time	MPG	MoBoo	Degree Discount	Single Discount	Degree
DBLP	100	0	87513.54 (1784.41)	85318.69 (1846.75)	94199.23 (1604.27)	**94576.77 (995.5)**	93577.21 (1648.73)
DBLP	250	0	89398.76 (1011.35)	86421.11 (1427.66)	**94648.08 (956.96)**	94093.91 (1609.46)	93649.4 (1154.01)
DBLP	100	2	84554.24 (2700.18)	82787.51 (1564.19)	**89040.37 (1922.22)**	88930.65 (1937.37)	88371.6 (1998.5)
DBLP	250	2	85123 (1528.91)	83169.94 (1165.33)	**89321.78 (1241.51)**	89117.05 (1273.56)	87952.33 (1496.25)
DBLP	100	5	74946.63 (3307.19)	76107.32 (2053.51)	**79130.22 (2571.46)**	78966.14 (2599.37)	78437.59 (2507.62)
DBLP	250	5	75191.35 (1787.43)	75044.26 (1581.74)	**78506.11 (1546.05)**	78386.73 (1639.76)	77234.03 (1613.53)
CA-CondMat	100	0	9095.72 (148.03)	8795.78 (188.43)	**9334.9 (128.99)**	9268.77 (138.18)	9262.39 (147.38)
CA-CondMat	250	0	9460.68 (125.48)	9096.58 (174.42)	**9816 (118.18)**	9646.13 (124.89)	9583.52 (113.73)
CA-CondMat	100	2	8564.83 (190.74)	8386.85 (205.89)	**8837.2 (174.96)**	8774.76 (180.65)	8740.71 (162.51)
CA-CondMat	250	2	8719.83 (158.04)	8523.77 (175.89)	**9277.7 (134.52)**	9123.6 (140.56)	9041.62 (140.75)
CA-CondMat	100	5	7407.06 (229.62)	7225.56 (274.2)	**7662.46 (205.08)**	7611.48 (228.84)	7568.37 (224.12)
CA-CondMat	250	5	7647.07 (168.09)	7303.5 (191.59)	**7902.9 (176.42)**	7730.11 (180.21)	7673.88 (207.03)
soc-Epinions1	100	0	29048.69 (128.96)	28913.33 (220.31)	29157.34 (173.28)	**29157.58 (158.24)**	29140.95 (164.02)
soc-Epinions1	250	0	29185.2 (130.19)	29058.39 (170.7)	**29264.5 (132.18)**	29232.3 (122.14)	29229.57 (124.89)
soc-Epinions1	100	2	22667.95 (918.36)	22449.7 (828.5)	**24110.08 (633.16)**	24085.77 (638.93)	24059.05 (654.23)
soc-Epinions1	250	2	21715.13 (537.88)	21551.11 (575.44)	**23809.58 (483.69)**	23712.39 (499.09)	23658.44 (491.44)
soc-Epinions1	100	5	**18239.53 (99.18)**	18156.3 (98.64)	18150.17 (111.5)	18150.91 (110.98)	18149.66 (109.48)
soc-Epinions1	250	5	**18450.6 (115.35)**	18353.61 (102.94)	18263.42 (100.41)	18265.78 (98.37)	18263.93 (97.66)

Furthermore, from Table 9, we see the typical pattern of heuristic performance decreasing as the burn-in time decreases. We also note that when the burn-in time is 5, MPG begins to perform better than the degree-based heuristics. However, this difference is not statistically significant, and introducing the controllable concept much later than time step 5 is unlikely to see further improvement, as the infection rate begins to rapidly drop.

#### 7.3.2 The Linear Threshold Model

Considering the LTM in real-world networks, we see similar results to using the ICM. For both CA-CondMat and soc-Epinions1, we see in Table 10 that the degree-based heuristics perform best, with no single heuristic consistently performing best. In CA-CondMat, the difference between the best and worst performing heuristics is small, but that performance gap becomes more significant in soc-Epinions1.

**Table 10:** Average infections for the target concept for real-world networks in the LTM and a r value of 2, with standard deviation in brackets, and the best performing heuristic in bold.

Network	Seed Set Size	Burn-in Time	MPG	MoBoo	Degree Discount	Single Discount	Degree
DBLP	100	0	6399.06 (5732.35)	**13359.45 (9594.71)**	11016.16 (10866.52)	12023.5 (11729.64)	12083.67 (10679.56)
DBLP	250	0	14704.2 (6859.42)	**28920.14 (8835.2)**	25148.84 (11144.31)	23303.53 (12201.94)	25001.57 (11340.43)
DBLP	100	2	9177.06 (7731.5)	**14668.27 (9377.16)**	10069.27 (10433.36)	10138.79 (10046.8)	8840.03 (9599.14)
DBLP	250	2	19446.17 (7415.16)	**27581.83 (8904.43)**	23737.61 (12903.52)	24298.5 (11996.1)	22546.35 (12018.63)
DBLP	100	5	6339.77 (5153.65)	**12746.51 (10001.78)**	9059.39 (11009.83)	8129.52 (8091.49)	11080.43 (11353.28)
DBLP	250	5	17552.01 (8182.74)	25799.76 (10232.7)	24628.93 (12525.97)	**26676.59 (12454.35)**	23194.25 (11309.94)
CA-CondMat	100	0	9648.98 (199.95)	9550.52 (168.1)	**9801.85 (170.92)**	9781.91 (157.99)	9797.23 (155.91)
CA-CondMat	250	0	10091.24 (155.91)	9813.31 (188.56)	**10387.1 (126.13)**	10354.84 (120.44)	10282.07 (142.14)
CA-CondMat	100	2	9430.33 (175.61)	9370.57 (155.09)	**9615.64 (163.11)**	9583.74 (165.9)	9588.57 (170.21)
CA-CondMat	250	2	9665.39 (169.82)	9571.63 (167.5)	**10124.72 (142.57)**	10089.18 (146.1)	10010.5 (137.3)
CA-CondMat	100	5	9240.24 (164.27)	9184.54 (166.09)	**9364.55 (171.51)**	9346.16 (163.72)	9339.59 (157.96)
CA-CondMat	250	5	9512.74 (161.65)	9458.72 (160.81)	**9890.69 (149.5)**	9854.11 (143.71)	9753.49 (144.21)
soc-Epinions1	100	0	18102.85 (479.51)	18353.21 (396.35)	**18894.11 (329.08)**	18815.04 (305.47)	18803.39 (362.84)
soc-Epinions1	250	0	18754.51 (485.88)	18827.79 (340.59)	**19594.75 (265.32)**	19464.95 (303.1)	19415.84 (304.76)
soc-Epinions1	100	2	17617.14 (453.54)	17760.02 (410.83)	18285.53 (365.25)	**18365.07 (335.23)**	18247.06 (352.5)
soc-Epinions1	250	2	18030.54 (426.84)	18181.44 (428.63)	19003.64 (327.77)	18859.19 (321.89)	**18865.09 (327.05)**
soc-Epinions1	100	5	17303.35 (470.66)	17287.62 (500.94)	17865.16 (375.64)	17855.96 (373.08)	**17930.77 (404.88)**
soc-Epinions1	250	5	17611.45 (495.2)	17596.77 (404.89)	**18486.33 (349.53)**	18342.47 (384.98)	18413.08 (314.61)

If we consider the DBLP network, we see that MoBoo is most often the best performing heuristic. With a smaller seed set, we see MoBoo consistently outperform all other heuristics, even as the burn-in time increases. However, with a larger seed set, we see the difference in performance lessen. This, combined with the general decrease in performance seen by the increase in burn-in time, leads to MoBoo being outperformed by the degree-based heuristics. We see that MPG performs significantly worse than the other heuristics in the DBLP network in the LTM. As noted before, in the LTM, a node will often require multiple active neighbours to become active. In a sparse network, such as DBLP, it becomes difficult for activations to occur and we never observe more than 9.1% of the network being activated.

### 7.4 Summary

MPG performed well in small-world networks, both in the ICM and LTM, only being significantly outperformed by MoBoo in the ICM when the burn-in time was 5 time steps. In scale-free networks we saw that degree-based heuristics are the most effective, but often the difference in performance was small. The characteristics of the network are clearly important, as highlighted by the experiments with real-world networks. The real-world networks had vastly different node counts, edge counts, diameters and densities. These factors all affect the best approach for indirect concept maximisation, with no heuristic consistently performing best.

The burn-in time and the increased efficiency of information spreading are the most significant factors impacting the performance of MPG when indirectly boosting the spread of a target concept. Within the LTM, we see the impact of burn-in time on heuristic performance diminished when compared to the ICM.

## 8 Results for indirect concept limitation

As with indirect concept maximisation, we evaluate the performance of MPG across a variety of network types. While the indirect influence limitation and indirect influence maximisation problems are similar, their opposite aims may change the characteristics that impact the performance of the various heuristics. We note that, except for MoBoo, all heuristics have previously been evaluated for the influence limitation problem using the ICM across small-world, scale-free and real-world networks in [9]. Most notably, the results for influence limitation in the ICM for small-world networks were the main focus of this previous work, while the heuristic evaluations in scale-free and real-world networks were much less detailed than presented here.

### 8.1 Small-world networks

We divide our discussion across the two influence spread models, as before. This allows us to highlight how the individual characteristics of those models affect indirect influence limitation.

#### 8.1.1 The Independent Cascade Model

In the context of the ICM, MPG significantly (*p* < 0.05) outperforms the other heuristics when there is no burn-in time, as shown in Table 11. This result is consistent across seed set sizes and network clustering coefficients. As in the indirect influence maximisation problem, we see minimal difference between the degree-based heuristics. We also note that, as before, network size and clustering coefficient has little effect on the performance of the different heuristics. Furthermore, we observe that when the focus is influence limitation, the MoBoo heuristic performs poorly. This is unexpected, as both MoBoo and MPG focus on utilising probable paths to calculate the expected gain of a node. However, in all cases, MoBoo performs worse than MPG for indirect influence limitation.

**Table 11:** Average infections for the target concept for small-world networks in the ICM, with no burn-in time and a r value of 0, with standard deviation in brackets, and the best performing heuristic in bold.

Network Size	Clustering Exponent	Seed Set Size	MPG	MoBoo	Degree Discount	Single Discount	Degree
25K	0.25	100 Seeds	**196.17 (16.91)**	222.11 (19.89)	218.26 (18.59)	218.71 (19.71)	218.13 (19.68)
25K	0.25	250 Seeds	**477.96 (29.03)**	548.4 (33.69)	527.64 (30.84)	527.4 (31.53)	528.88 (30.69)
25K	0.25	500 Seeds	**924.11 (39.33)**	1072.08 (46.75)	1005.3 (40.94)	1007.34 (40.4)	1007.11 (39.46)
25K	0.75	100 Seeds	**195.53 (17.04)**	221.32 (19.44)	218 (19.33)	217.75 (17.96)	217.7 (18.56)
25K	0.75	250 Seeds	**476.78 (28.14)**	545.96 (31.93)	527.74 (30.88)	526.58 (30.11)	528.58 (30.12)
25K	0.75	500 Seeds	**926.95 (35.66)**	1070.57 (44.35)	1008.87 (40.34)	1007.51 (39.1)	1006.26 (38.15)
50K	0.25	100 Seeds	**199.03 (18.17)**	222.24 (19.48)	220.93 (19.62)	220.66 (19.77)	220.93 (19.79)
50K	0.25	250 Seeds	**487.13 (28.19)**	552.1 (33.77)	541.93 (32.733)	541.96 (33.94)	541.58 (33.92)
50K	0.25	500 Seeds	**954.39 (38.48)**	1090.49 (46.42)	1053.61 (43.77)	1053.82 (43.87)	1052.27 (44.89)
50K	0.75	100 Seeds	**197.37 (17.55)**	222.72 (20.26)	219.29 (19.54)	220.23 (19.5)	220.35 (19.8)
50K	0.75	250 Seeds	**485.69 (30.17)**	551.98 (33.97)	540.7 (32.74)	541.18 (31.91)	541.1 (32.48)
50K	0.75	500 Seeds	**954.79 (38.74)**	1089.3 (46.47)	1055.72 (43.79)	1054.37 (43.68)	1054.72 (41.29)
100K	0.25	100 Seeds	**197.88 (19.52)**	222.63 (20.48)	221.83 (20.93)	221.8 (20.84)	221.45 (19.87)
100K	0.25	250 Seeds	**491.21 (29.61)**	553.77 (34.34)	548.68 (33.08)	548.47 (32.84)	548.44 (33.39)
100K	0.25	500 Seeds	**974.5 (42.95)**	1100.18 (48.56)	1079.57 (47.63)	1080.02 (47.32)	1079.74 (45.82)
100K	0.75	100 Seeds	**196.83 (17.52)**	222.27 (19.85)	221.07 (19.41)	221.72 (19.33)	221.68 (19.6)
100K	0.75	250 Seeds	**490.74 (28.78)**	554.43 (33.25)	549.35 (33.95)	549.02 (32.9)	549.17 (33.46)
100K	0.75	500 Seeds	**971.04 (40.17)**	1100.13 (46.48)	1079.89 (44.8)	1079.78 (46.07)	1079.64 (46)
250K	0.25	100 Seeds	**198.95 (16.7)**	222.66 (20.36)	222.46 (19.37)	222.46 (19.37)	222.49 (19.38)
250K	0.25	250 Seeds	**494.45 (29.46)**	556.15 (33.65)	553.33 (33.27)	553.24 (33.29)	553.19 (33.27)
250K	0.25	500 Seeds	**983.66 (41.77)**	1105.83 (47.06)	1097.39 (46.42)	1097.61 (46.29)	1097.72 (46.08)
250K	0.75	100 Seeds	**198.86 (17.97)**	222.96 (20.74)	222.9 (20.52)	222.9 (20.52)	222.9 (20.51)
250K	0.75	250 Seeds	**494.65 (29.08)**	555.11 (33.59)	552.45 (34.54)	552.46 (34.54)	552.57 (34.25)
250K	0.75	500 Seeds	**984.64 (45.35)**	1106.14 (48.2)	1096.94 (48.65)	1096.59 (48.18)	1097.19 (48.26)

**Fig 9:**
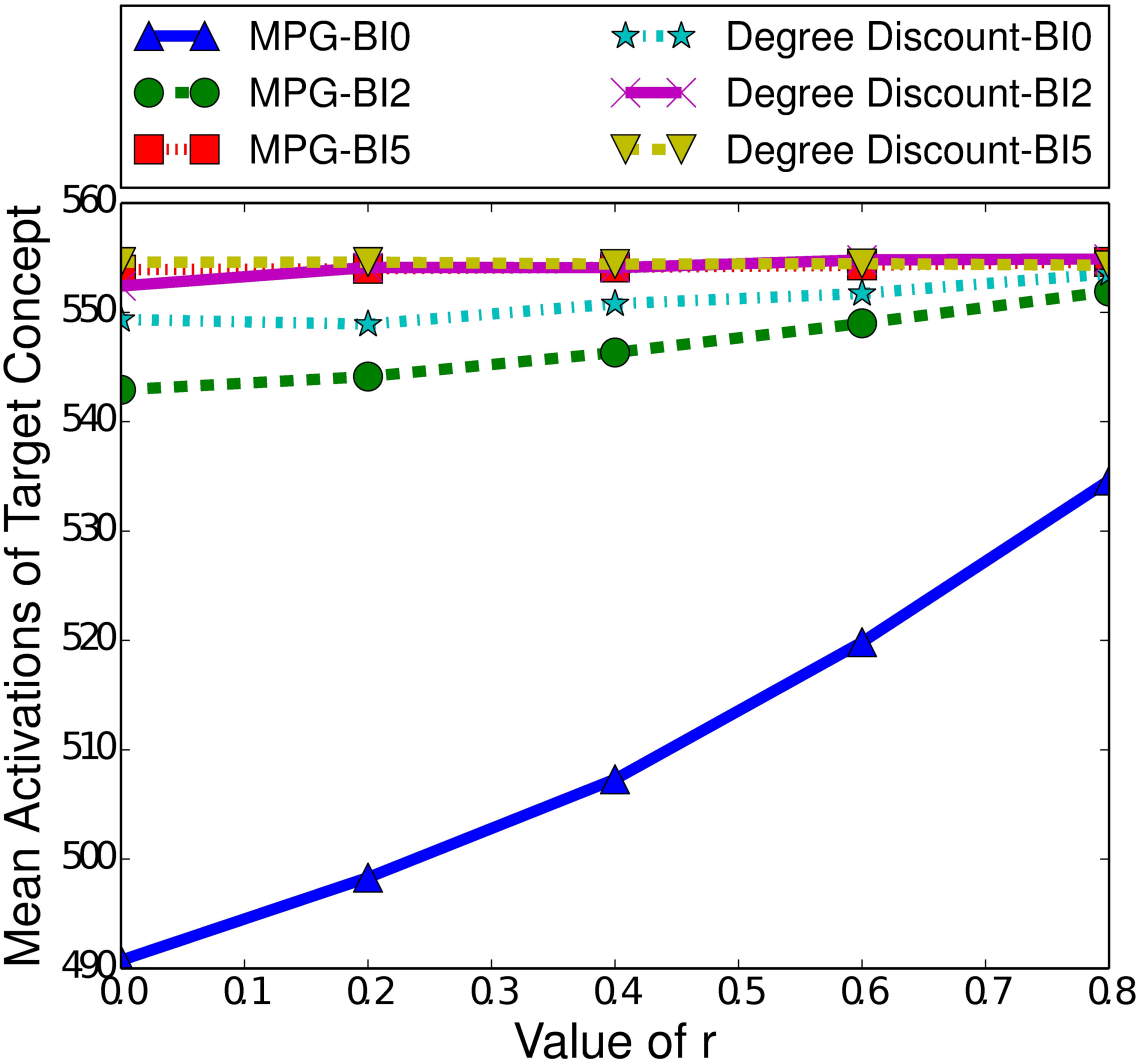
Mean activations of the target concept given the heuristic used to select the inhibiting concept in the ICM, for small-world networks of 100000 nodes, with a clustering coefficient of 0.75, a seed set of 250 nodes and a variety of burn-in (BI) times.

As Fig 9 demonstrates, as the burn-in time increases the performance of MPG becomes significantly worse. The performance drop from a burn-in time of 2 to 5 is much less than that from 0 to 2, which is to be expected. The majority of spreading for a concept occurs in the first 2 time steps, meaning that the ability of the controllable concept to significantly limit the spread of the target concept is diminished. At the highest burn-in time, there is no statistically significant difference between the performance of all heuristics (*p* > 0.5), and even the strength of the relationship between concepts has little effect. This is in contrast to MPG with a burn-in time of 0 where we see that as the relationship strength decreases, becoming more inhibiting, the performance of MPG noticeably increases.

#### 8.1.2 The Linear Threshold Model

Table 12 shows that MPG maintains its superior performance in small-world networks in the LTM. Overall, we can see that the number of target concept activations decreases when compared to the same parameters in the ICM. This is intuitive since the LTM is threshold based. A node with the controllable concept active will have all incoming influence decreased, and will exert decreased influence. This makes the node more difficult to activate and it will contribute less to the activation of other nodes. Furthermore, the controllable concept can also spread through the local area, making it difficult to activate any node in the neighbourhood.

**Table 12:** Average infections for the target concept for small-world networks in the LTM, with no burn-in time and a r value of 0, with standard deviation in brackets, and the best performing heuristic in bold.

Network Size	Clustering Exponent	Seed Set Size	MPG	MoBoo	Degree Discount	Single Discount	Degree
50K	0.25	250 Seeds	**338.34 (16.62)**	597.54 (43.81)	593.77 (32.34)	593.79 (32.15)	593.71 (32.34)
50K	0.25	500 Seeds	**671.55 (23.95)**	1167.26 (52.18)	1135.33 (45.42)	1135.05 (45.49)	1135.21 (45.46)
50K	0.75	250 Seeds	**598.06 (17.99)**	337.14 (38.39)	589.76 (37.41)	589.64 (37.01)	589.82 (37.4)
50K	0.75	500 Seeds	**667.1 (23.78)**	1174.07 (47.61)	1135.17 (46.47)	1134.7 (46.22)	1135.33 (46.56)
100K	0.25	250 Seeds	**340.05 (17.82)**	603.96 (39.21)	601.93 (43.73)	602.08 (43.73)	602.14 (43.64)
100K	0.25	500 Seeds	**678.01 (26.35)**	1198.49 (53.87)	1177.49 (55.81)	1177.73 (55.25)	1177.16 (55.59)
100K	0.75	250 Seeds	**339.44 (20.07)**	611.12 (36.25)	598.64 (43.77)	598.62 (43.79)	598.67 (43.74)
100K	0.75	500 Seeds	**676.32 (24.24)**	1206 (49.03)	1174.25 (53.27)	1174.56 (53.05)	1174.3 (53.24)
250K	0.25	250 Seeds	**340.26 (16.74)**	603.14 (41.04)	606 (38.57)	605.99 (38.58)	605.99 (38.59)
250K	0.25	500 Seeds	**677.78 (24.19)**	1205.73 (54.79)	1202.57 (57.64)	1202.57 (57.54)	1202.54 (57.66)
250K	0.75	250 Seeds	**340.19 (19.12)**	616.38 (35.81)	608.34 (36.77)	608.35 (36.76)	608.34 (36.77)
250K	0.75	500 Seeds	**681.2 (26.89)**	1216.52 (57.04)	1213.9 (57.54)	1213.82 (57.59)	1213.91 (57.53)

**Fig 10:**
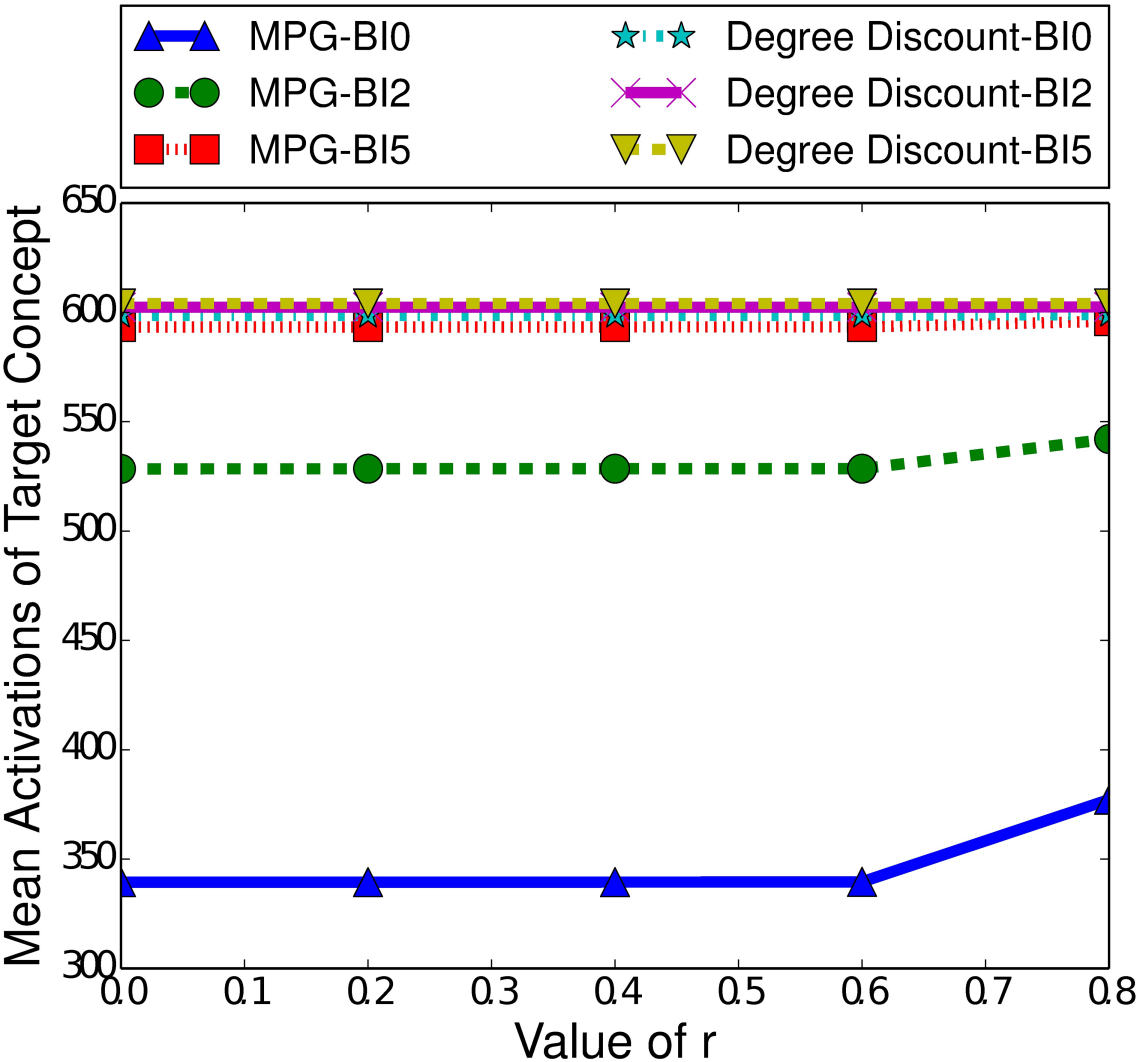
Mean activations of the target concept given the heuristic used to select the inhibiting concept in the LTM, for small-world networks of 100000 nodes, with a clustering coefficient of 0.75, a seed set of 250 nodes and a variety of burn-in (BI) times.

Unlike in the problem of indirect influence maximisation, we do not see an increased burn-in time result in an increase to the performance of MPG in the LTM. As Fig 10 shows, increasing the burn-in time has a similar impact as in the ICM. The primary difference is that the strength of the relationship is less important in the LTM, as it is only at the weakest inhibiting relationship that we see a change in the performance of MPG.

### 8.2 Scale-free networks

In the indirect influence maximisation problem, we saw that the existence of hub nodes resulted in the heuristics that considered relationships, namely MPG and MoBoo, becoming less effective. We now consider their effectiveness for indirect influence limitation.

#### 8.2.1 The Independent Cascade Model

Observing scale-free networks in the ICM we see that all non-random heuristics perform at a similar level when the burn-in time is 0, as shown in Table 13, aside from MoBoo which consistently performs the worst, by a significant margin. Unlike when considering indirect influence maximisation, we see that seed set size, in addition to network size and clustering exponent, all affect the performance of the heuristics. For indirect influence limitation, increasing the seed set size of the target concept will naturally result in more activations. As such, we see the performance of the influence limitation heuristics worsen. Similarly, increasing the size of the network or the number of edges added during creation again aids the spread of the target concept and increases the difficulty in limiting its spread.

**Table 13:** Average infections for the target concept in the ICM for scale-free networks, with no burn-in time, and a r value of 0, with standard deviation in brackets, and the best performing heuristic in bold.

Network Size	Edges Added	Seed Set Size	MPG	MoBoo	Degree Discount	Single Discount	Degree
25K	4	250	**679.91 (64.34)**	3839.63 (315.33)	693.05 (61.13)	692.94 (58.51)	689.08 (55.4)
25K	8	250	1404.89 (105.39)	9616.39 (460.44)	1380.54 (100.03)	**1369.26 (100.47)**	1383.4 (100.95)
25K	4	500	1124.03 (62.16)	4321.21 (238.29)	1096.64 (51.43)	**1093.99 (48.98)**	1108.11 (58.16)
25K	8	500	2058.38 (97.95)	9749.37 (318.37)	**1967.79 (98.03)**	1967.88 (101.79)	1984.56 (97.13)
50K	4	250	**857.49 (107.28)**	7351.64 (663.92)	887.24 (91.61)	892.04 (95.65)	893.19 (104.35)
50K	8	250	1897.95 (171.95)	19209.67 (897.6)	**1894.35 (149.38)**	1906.72 (163.82)	1912.83 (166.48)
50K	4	500	**1349.77 (89.88)**	7628.29 (460.65)	1374.86 (87.5)	1374.88 (81.7)	1372.88 (80.9)
50K	8	500	2783.71 (136.62)	19343.62 (587.31)	2712.33 (129.78)	**2708.49 (129.67)**	2718.76 (135.2)
100K	4	250	**1148.82 (178.87)**	13719.66 (1291.69)	1155.54 (155.96)	1159.02 (137.81)	1165.08 (137.1)
100K	8	250	2676.92 (259.14)	38251.34 (2161.68)	2654.55 (212.66)	2639.1 (216.7)	2656.25 (206.13)
100K	4	500	**1711.19 (145.84)**	14602.97 (892.33)	1776.05 (140.22)	1770.05 (134.68)	1776.66 (134.13)
100K	8	500	3813.03 (223.98)	38565.04 (1422.69)	3788.38 (92.36)	3763.92 (173.45)	**3739.02 (195.9)**

We note that the increased efficiency with which information can spread in scale-free networks naturally makes inhibition less effective. MPG focuses on finding nodes with a high number of expected activations, that are likely to be activated by the target concept, typically selecting nodes of high degree or the neighbours of those nodes. Due to the degree distribution of scale-free networks, there are nodes with a degree exceeding 500 in a 25000 node network. If the target concept is activated on one of these high degree nodes, it can then efficiently spread to a large proportion of the network, the majority of which will not have the controllable concept active. This means that the impact of the controllable concept becomes negligible.

**Fig 11:**
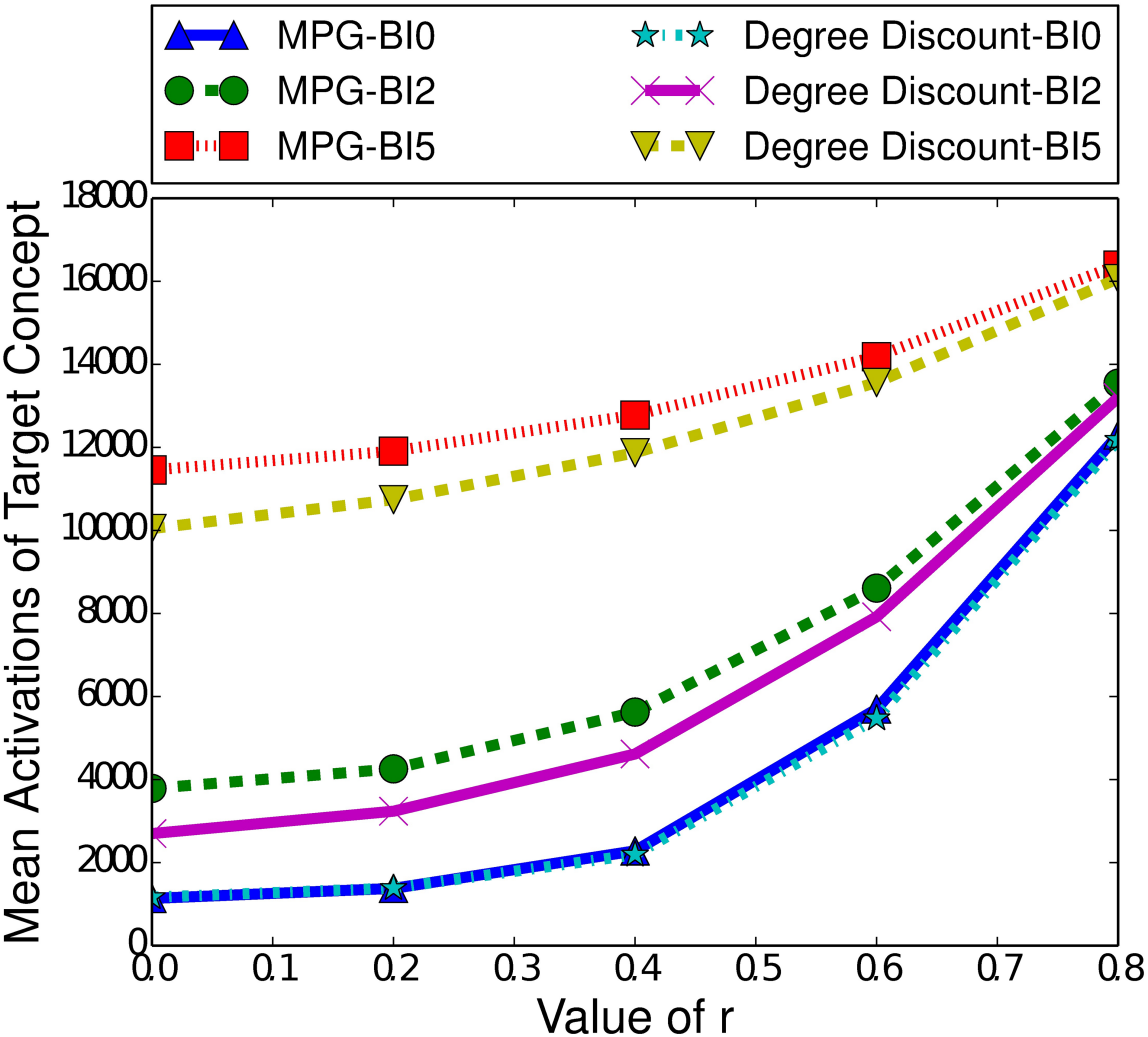
Mean activations of the target concept given the heuristic used to select the inhibiting concept in the ICM, for scale-free networks of 100000 nodes, with 4 edges added each round, a seed set of 250 nodes and a variety of burn-in (BI) times.

In terms of the effect of the burn-in time, Fig 11 shows that, at a burn-in time of 0, both heuristics perform at a similar level. Then, as the burn-in time increases, we see that degree discount begins to out-perform MPG. This is to be expected, due to MPG’s noted sensitivity to the burn-in time.

#### 8.2.2 The Linear Threshold Model

Comparing influence limitation in the LTM, shown in Table 14, to the ICM, we see that for scale-free networks there are uniformly more activations in the LTM. Again, the heuristics all perform similarly, with MoBoo being the only outlier. Furthermore, MPG does not perform as well in networks with 4 edges added as it did in the ICM. As in the ICM, we see that all of the network characteristics can affect the general performance of the heuristics but not drastically enough to make one perform significantly better than the rest.

**Table 14:** Average infections for the target concept in the LTM for scale-free networks, with no burn-in time, a seed set of 500 and a r value of 0, with standard deviation in brackets, and the best performing heuristic in bold.

Network Size	Edges Added	Seed Set Size	MPG	MoBoo	Degree Discount	Single Discount	Degree
25K	4	250	744.32 (148.88)	1975.86 (166.01)	747.32 (66.28)	**739.42 (56.13)**	741.6 (57.43)
25K	8	250	2725.67 (150.86)	4857.8 (155.48)	**2522.44 (135.4)**	2530.31 (131.7)	2534.95 (131.74)
25K	4	500	**1013.95 (82.8)**	2407.01 (136.35)	1120.0 (47.31)	1108.19 (46.43)	1109.29 (47.61)
25K	8	500	2824.46 (133.05)	5030.96 (145.49)	**2644.97 (126.45)**	2648.17 (122.32)	2650.47 (121.33)
50K	4	250	1340.5 (258)	3424.76 (262.39)	1015.36 (123.61)	**1004.03 (115.99)**	1004.2 (116.86)
50K	8	250	5486.35 (247.16)	9405.87 (223.6)	**5084.67 (212.84)**	5104.22 (204.07)	5095.78 (206.88)
50K	4	500	1447.93 (169.974)	3932.64 (237.26)	1453.77 (86.65)	**1439.89 (85.69)**	1440.65 (88.87)
50K	8	500	5444.5 (215.11)	9720.97 (218.6)	**5030.87 (214.46)**	5074.77 (201.8)	5072.84 (202.59)
100K	4	250	2694.95 (445.87)	6305.49 (462.03)	**1660.87 (233.67)**	1626.59 (214.26)	1626.21 (218.29)
100K	8	250	11145.02 (374.08)	18498.89 (456.31)	**10330.67 (337.47)**	10349.4 (326.85)	10345.56 (333.45)
100K	4	500	2620.16 (427.85)	6848.26 (401.36)	2059.72 (169.5)	2039.24 (165.09)	**2035.81 (165.04)**
100K	8	500	10917.9 (320.56)	18748.83 (425.15)	10082.23 (310.57)	**10070.41 (303.86)**	10076.19 (307.84)

**Fig 12:**
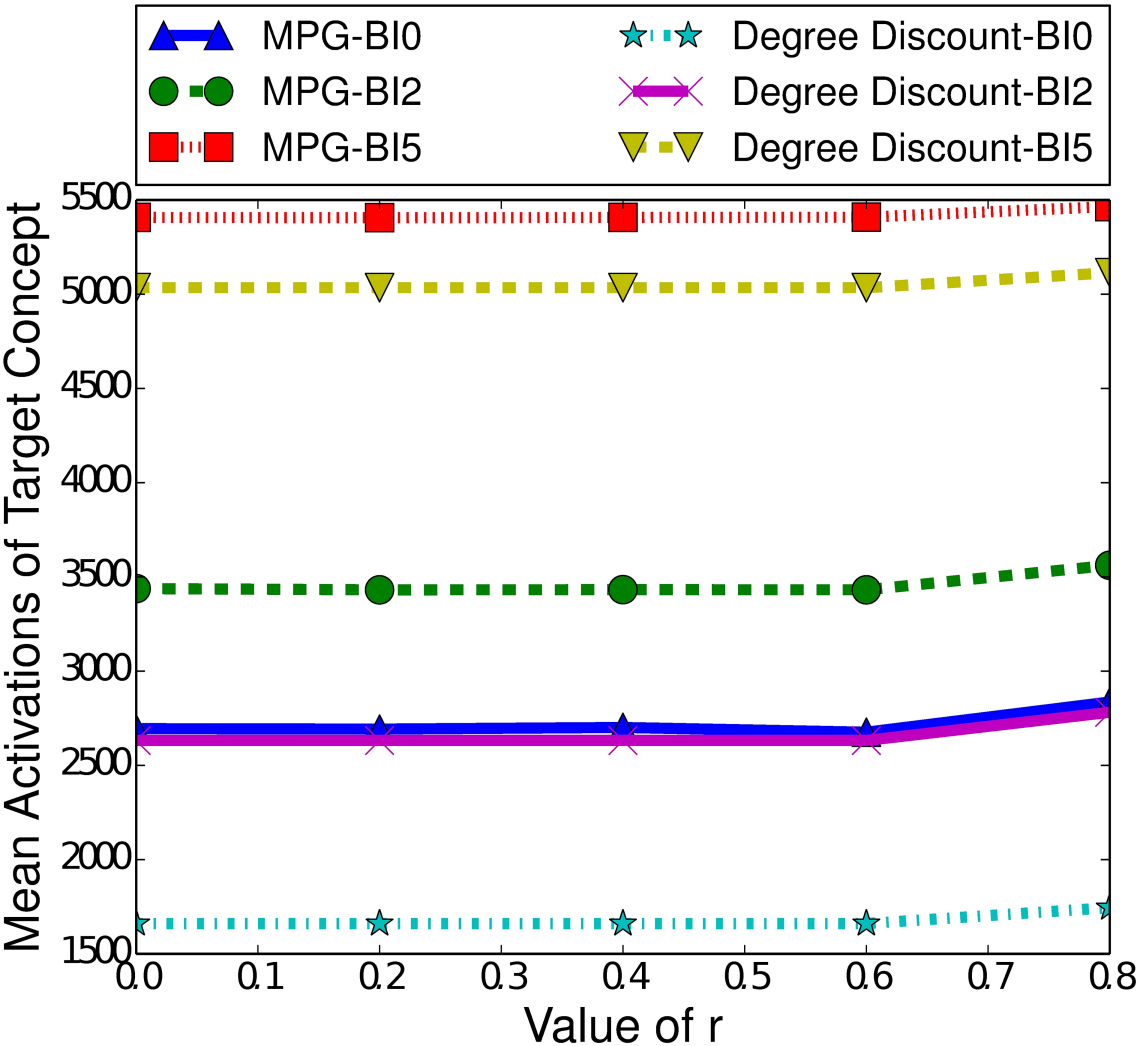
Mean activations of the target concept given the heuristic used to select the inhibiting concept in the LTM, for scale-free networks of 100000 nodes, with 4 edges added each round, a seed set of 250 nodes and a variety of burn-in (BI) times.

Considering the impact of the burn-in time on performance in the LTM, Fig 12 shows that, unlike in the ICM, increasing the burn-in time decreases the difference in performance. In all cases, the degree discount heuristic performs better, but the difference noticeably decreases between each different burn-in time. Furthermore, the decreased significance of relationship strength in the LTM is also displayed.

### 8.3 Real-world networks

We saw that the properties of the real-world networks had a significant impact on the performance of the various heuristics when studying the indirect influence maximisation problem. We now consider how these properties affect the heuristics in the indirect influence limitation problem.

#### 8.3.1 The Independent Cascade Model

When considering the ICM, we see that no single heuristic consistently performs the best. Table 15 shows that, most often, degree discount is the best performing heuristic, but can be outperformed by MPG and single discount. Overall, the performance of all the heuristics is inconsistent.

**Table 15:** Average infections for the target concept for real-world networks in the ICM and a r value of 0, with standard deviation in brackets, and the best performing heuristic in bold.

Network	Seed Set Size	Burn-in Time	MPG	MoBoo	Degree Discount	Single Discount	Degree
DBLP	100	0	**695.68 (341.04)**	48038.47 (430.79)	1076.65 (335.48)	1102.83 (318.76)	1161.06 (389.88)
DBLP	250	0	**1117.76 (252.22)**	48328.54 (477.65)	1710.72 (283.72)	1773.45 (281.41)	1954.52 (381.47)
DBLP	100	2	4077.98 (2256.43)	48038.47 (430.79)	**4001.55 (1516.67)**	4168.8 (1651.04)	4439.05 (1621)
DBLP	250	2	6074.14 (1483.02)	48327.88 (477.58)	**5352.16 (1123.13)**	5516.6 (1115.5)	6291.91 (1337.28)
DBLP	100	5	24321.11 (5449.5)	48003.03 (512.08)	**22947.29 (5363.24)**	23275.05 (5270.99)	24046.62 (5211.48)
DBLP	250	5	28984.87 (2416)	48267.1 (525.59)	**26803.7 (3058.68)**	27189.96 (2928.57)	28698.01 (2998.38)
CA-CondMat	100	0	**367.31 (70.26)**	5203.62 (111.53)	415 (63.26)	439.66 (74.06)	440.62 (82.92)
CA-CondMat	250	0	722.86 (75.84)	5393.57 (126.92)	**676.51 (55.69)**	699.74 (69.06)	735.29 (65.65)
CA-CondMat	100	2	1498.91 (330.4)	5204.2 (117.17)	**1138.86 (224.28)**	1139.13 (223.63)	1158.96 (225.85)
CA-CondMat	250	2	2265.92 (236.91)	5400.62 (109.69)	1725.59 (181.25)	**1689.67 (174.33)**	1708.92 (177)
CA-CondMat	100	5	4027.35 (268.74)	5194.55 (114.85)	**3944.87 (317.05)**	3992.39 (318.18)	4020.92 (323.61)
CA-CondMat	250	5	4472.88 (163.6)	5397.4 (112.96)	**4463.39 (161.51)**	4524.04 (170.46)	4573.53 (172.74)
soc-Epinions1	100	0	469.36 (123.01)	18097.45 (93.6)	324.14 (71.84)	**320.57 (72.06)**	324.51 (71.96)
soc-Epinions1	250	0	938.72 (162.6)	18236.71 (101.23)	**575.31 (73.9)**	580.86 (71.29)	584.92 (71.17)
soc-Epinions1	100	2	13582.4 (1332.62)	18096.5 (94.56)	**10448.56 (1300.4)**	10488.88 (1325.74)	10538.08 (1310.38)
soc-Epinions1	250	2	15401.64 (529.79)	18235.43 (102.62)	**11681.92 (886.95)**	11771.71 (908.25)	11856.88 (908.16)
soc-Epinions1	100	5	**18015.98 (101.96)**	18096.8 (93.66)	18087.44 (107.83)	18087.05 (108)	18086.61 (109)
soc-Epinions1	250	5	**18146.03 (107.81)**	18237.94 (101.68)	18214.15 (99.24)	18216.01 (98.69)	18215.62 (99.03)

In the DBLP network, we see that MPG performs significantly better than the other heuristics, when the burn-in time is 0. The sparser nature of this network, as previously discussed, means that it is difficult for concepts to spread, which makes the local targeting of MPG more effective at inhibition. This advantage is soon lost, and as the burn-in time increases MPG’s relative performance decreases, as we have seen throughout this analysis. It becomes comparable to the degree-based heuristics at a burn-in time of 2, and is noticeably outperformed by degree discount at higher burn-in times.

In the CA-CondMat network, we see MPG perform best for small seed sets and burn-in time of 0, then begin to perform noticeably worse than the degree-based heuristics at larger burn-in times. In particular, the distance between the performance of the degree-based heuristics and MPG is greatest when the burn-in time is 2. At a burn-in time of 5, it is likely that the target cascade has performed the majority of its spreading and so no heuristic can make a significant difference in the final number of infections. At a burn-in time of 2, however, MPG’s strategy of targeting local areas is clearly ineffective compared to spreading the controllable concept as widely as possible. Furthermore, we see very similar behaviour in the soc-Epinions1 network which, as we have already discussed, has similar properties to CA-CondMat.

#### 8.3.2 The Linear Threshold Model

In the LTM, we see similar inconsistency across the real-world networks as we observed in the ICM. The degree-based heuristics often perform the best but, as seen in Table 16, no single heuristic is clearly superior.

**Table 16:** Average infections for the target concept for real-world networks in the LTM and a r value of 0, with standard deviation in brackets, and the best performing heuristic in bold.

Network	Seed Set Size	Burn-in Time	MPG	MoBoo	Degree Discount	Single Discount	Degree
DBLP	100	0	**150.59 (29.25)**	818.75 (1126.71)	387.14 (238.04)	389.5 (289.51)	382.83 (268.77)
DBLP	250	0	**371.0 (42.34)**	1819.74 (1285.6)	811.61 (281.73)	823.26 (262.54)	869.1 (300.75)
DBLP	100	2	**426.82 (409.95)**	719.04 (839.6)	542.21 (394.64)	502.75 (546.26)	528.24 (449.21)
DBLP	250	2	**813.53 (297.86)**	1652.41 (951.02)	1092.62 (395.94)	1028.39 (398.37)	1021.64 (410.66)
DBLP	100	5	661.88 (725.68)	724.97 (738.17)	**596.58 (577.28)**	628.24 (639.14)	716.86 (672.03)
DBLP	250	5	**1411.78 (819.06)**	1709.75 (1136.8)	1520.83 (974.53)	1523.21 (1014.14)	1462.79 (849.88)
CA-CondMat	100	0	1898.87 (152.3)	3623.61 (131.22)	1901.43 (115.18)	**1894.62 (142.38)**	1899.95 (137.76)
CA-CondMat	250	0	2018.13 (139.6)	3779.66 (134.02)	1911.76 (135.02)	**1892.12 (123.51)**	1952.11 (112.29)
CA-CondMat	100	2	2783.63 (161.31)	3669.68 (122.79)	2480.05 (145.27)	**2475.29 (156.10)**	2497.97 (170.58)
CA-CondMat	250	2	3124.96 (118.76)	3886.95 (113.66)	**2767.95 (129.35)**	2770.9 (129.06)	2802.03 (146.61)
CA-CondMat	100	5	3547.85 (127.73)	3713.63 (121.71)	3549.1 (118.28)	3527.02 (125.7)	3538.62 (111.67)
CA-CondMat	250	5	3824.05 (115.21)	3929.5 (100.65)	3781.63 (118.37)	**3763.88 (112.04)**	3797.15 (107.21)
soc-Epinions1	100	0	3530.42 (231.68)	6437.22 (227.46)	3425.16 (164.25)	**3381.99 (177.01)**	3385.02 (179.73)
soc-Epinions1	250	0	3604.14 (205.87)	6574.88 (214.73)	**3399.9 (153.49)**	3427.44 (145.38)	3443.53 (166.07)
soc-Epinions1	100	2	4965.82 (279.78)	6416.25 (222.03)	4519.49 (274.53)	**4475.01 (244.86)**	4573.11 (244.53)
soc-Epinions1	250	2	5199.34 (264.67)	6581.87 (218.31)	**4565.49 (215.04)**	4695.33 (247.55)	4689.41 (264.83)
soc-Epinions1	100	5	6317.56 (206.88)	6427.41 (237.13)	6281.77 (193.37)	6265.21 (211.03)	**6251.91 (215.15)**
soc-Epinions1	250	5	6518.58 (212.89)	6578.71 (210.71)	6473.05 (222.3)	6467.63 (204.79)	**6459.15 (225.75)**

We also observe how the LTM is less affected by burn-in time in the performance of MPG in the DBLP network. When compared to the ICM, we see that in the LTM, MPG maintains superior performance in the majority of environments, even as the burn-in time increases. However, the DBLP network results in all heuristics having significantly higher standard deviations than in other networks. As such, it is difficult to confidently say that MPG is the best choice for indirect influence limitation in the DBLP network, especially at higher burn-in times.

For both CA-CondMat and soc-Epinions1, we see the same behaviour in the ICM. At a burn-in time of 5, all heuristics perform similarly and MPG has comparatively the worst performance when the burn-in time is 2.

### 8.4 Summary

Overall, we see that MPG performs best in small-world networks, with no burn-in time, for both influence spread models. In these networks, MPG consistently and significantly outperforms the degree-based heuristics. With a high burn-in time, all heuristics performed at a similar level, due to the inhibiting concept being introduced as the target concept cascade is ending. In scale-free networks, we see no heuristic performing significantly better than others. Similarly, in real-world topologies we see that none of the heuristics consistently maintains superior performance when compared to the others.

## 9 Computational Performance

We present a brief discussion on the runtime performance of the three most effective heuristics, namely degree discount, MPG and MoBoo. These three heuristics require different levels of information and perform varying levels of calculation, and it is important to evaluate how the runtime of each heuristic scales as the size of the network grows. The results of our tests can be seen in Table 17.

**Table 17:** Average execution time of heuristics to select 250 seeds in various graph types. Averages from 100 runs. Times are in seconds. All runs performed on an Intel^®^ Core™ i5-4590 CPU with 4 3.30 GHz cores. For small-world (SW) networks, the secondary characteristic is their clustering exponent, for scale-free (SF) networks, secondary characteristic refers to the number of edges added during each creation step.

Network Size	Network Type	Secondary Characteristic	Degree Discount Time	MPG Time	MoBoo Time
25000	SW	0.25	2.54	1.41	7.46
50000	SW	0.25	6.2	1.65	16.41
100000	SW	0.25	15.83	1.83	32.27
25000	SW	0.75	2.14	1.11	6.48
50000	SW	0.75	5.53	1.32	14.22
100000	SW	0.75	14.12	1.53	28.96
25000	SF	4	2.81	30.93	16.4
50000	SF	4	6.73	48.87	62.08
100000	SF	4	17.12	90.16	140.82
25000	SF	8	3.36	224.11	21.04
50000	SF	8	7.05	429.01	60.13
100000	SF	8	16.56	757.58	181.39

Degree discount has the most consistent execution time, which increases slowly as the size of the network increases. Of the three heuristics, we see that degree discount is the only one not to have its execution time increase significantly when used in scale-free networks, remaining comparable to its execution time in small-world networks.

In small-world networks, MoBoo’s execution time increases at a similar rate to degree discount, but with higher initial values. In scale-free networks, MoBoo’s execution time increases at a higher rate, as both the number of nodes and edges increases. Furthermore, in small-world networks MoBoo consistently has the worst execution time, and in scale-free networks it increases at the highest rate, although it is generally faster than MPG.

MPG is significantly affected by the type of network it is applied to. In small-world networks, MPG often executes fastest and scales with the slowest rate. Since MPG best manipulates the spread of a concept within small-world networks, this means that it runs very quickly in the best scenario for its use. In scale-free networks, MPG scales poorly, especially in the more connected networks with 8 edges added for each node. The aim of MPG is to limit exploration to a local area to prevent the need to search the entire network multiple times. However, in scale-free networks, the presence of hub nodes means that many nodes contain the majority of the network within their 2-hop neighbourhood. As we can see, the execution time of MPG suffers accordingly. The high execution time, and low effectiveness, means that we should avoid the use of MPG in scale-free networks, especially when compared to degree discount. Degree discount has been shown to be at least as effective as MPG in scale-free networks, with a fraction of the execution time.

## 10 Conclusion

In this paper, we have defined the indirect influence maximisation and indirect influence limitation problems, in which a controllable concept is used to indirectly affect the spread of a target concept. We proposed the MPG heuristic as a method for addressing both indirect influence maximisation and indirect influence limitation, and evaluated its effectiveness against previously used influence maximisation heuristics. For both problems, we evaluated MPG in a variety of small-world, scale-free and real-world networks for both the ICM and LTM.

In both the indirect influence maximisation and indirect influence limitation problems, we found that in the ICM MPG performs effectively in small-world networks with a burn-in time of 0. In scale-free networks and any environment with no burn-in time the difference in performance between the heuristics begins to disappear. The MoBoo heuristic was the only heuristic examined that could consistently outperform MPG, but only in small-world networks with a high burn-in time when attempting to boost the target concept. However, unlike MPG, we saw that MoBoo could not adapt to the problem of influence limitation. Overall, we see that MPG is the best performing heuristic in small-world networks, with influence manipulation in scale-free networks seeming to be much less effective. In some cases, MPG performs better in the LTM, due to the decreased significance of burn-in time when compared to the ICM.

In particular, we see that MPG is most suited for limiting the spread of a concept when the controllable concept can be introduced at the same time, or very soon after, the target concept. For example, if a network is being observed and an undesirable concept, such as an epidemic or competing product, begins to spread in one area, we may focus on a local area nearby that we expect to be exposed to this concept. MPG can be used in this instance to introduce a controllable concept to a local area at time close to when we expect the undesirable concept to reach that area. In the real-world it is typically impossible to introduce a new concept at the exact time as an undesirable concept begins to spread, but it is possible to predict areas that may be exposed to a spreading concept. If we can introduce our controllable concept at that time, then MPG provides an effective method to limit the spread of the undesirable concept to that local area. In a large network, this can be applied to several areas simultaneously, allowing for a wider area of the network to be protected. Ideally, the local network we choose will exhibit small-world properties, since hub nodes lower the effectiveness of indirect concept manipulation in general.

When considering the real-world networks, we saw that the differences in their parameters result in different performances for each heuristic, though none was consistently superior. This further highlights the impact of network parameters, making the development of a general indirect influence manipulation heuristic difficult.

In the future, we will investigate the use of MPG to manipulate influence spread in other spreading models. For example, we will consider the SIS model and a more general version of the SIR model [12, 13]. These provide variations in how a concept may spread through a network, and the ability to manipulate a concept’s spread in these models will allow for investigation of problems focused on tackling epidemics and disease spread. In addition, we also wish to investigate the performance of MPG in more complex network environments, including in multi-layer networks, representing the different social networks an individual may belong to.

## References

[1] Kempe D, Kleinberg J, Tardos É. Maximizing the spread of influence through a social network. In: Proceedings of the 9th ACM SIGKDD International Conference on Knowledge Discovery and Data Mining; 2003. p. 137–146.

[2] Christakis NA, Fowler JH. The collective dynamics of smoking in a large social network. New England journal of medicine. 2008;358(21):2249–2258. doi: 10.1056/NEJMsa0706154 18499567 10.1056/NEJMsa0706154 PMC2822344

[3] Goldenberg J, Libai B, Muller E. Using Complex Systems Analysis to Advance Marketing Theory Development. Academy of Marketing Science Review. 2001;9(3):1–18.

[4] Chen W, Wang Y, Yang S. Efficient influence maximization in social networks. In: Proceedings of the 15th ACM SIGKDD International Conference on Knowledge Discovery and Data Mining; 2009. p. 199–208.

[5] Goyal S, Kearns M. Competitive contagion in networks. In: Proceedings of the 44th annual ACM Symposium on Theory of Computing; 2012. p. 759–774.

[6] He X, Song G, Chen W, Jiang Q. Influence Blocking Maximization in Social Networks under the Competitive Linear Threshold Model. In: Proceedings 12th SIAM International Conference on Data Mining; 2012. p. 463–474.

[7] Sanz J, Xia CY, Meloni S, Moreno Y. Dynamics of interacting diseases. Physical Review X. 2014;4(4):041005 doi: 10.1103/PhysRevX.4.041005

[8] Archbold J, Griffiths N. Maximising influence in non-blocking cascades of interacting concepts. In: International Workshop on Multi-Agent Systems and Agent-Based Simulation. Springer; 2015. p. 173–187.

[9] Archbold J, Griffiths N. Limiting Concept Spread in Environments with Interacting Concepts. In: Proceedings of the 16th Conference on Autonomous Agents and MultiAgent Systems; 2017. p. 1332–1340.

[10] Wang S, Zhao X, Chen Y, Li Z, Zhang K, Xia J. Negative influence minimizing by blocking nodes in social networks. In: Proceedings of the 17th AAAI Conference on Late-Breaking Developments in the Field of Artificial Intelligence; 2013. p. 134–136.

[11] Fan L, Lu Z, Wu W, Thuraisingham B, Ma H, Bi Y. Least cost rumor blocking in social networks. In: Proceedings of the 33rd IEEE International Conference on Distributed Computing Systems; 2013. p. 540–549.

[12] Chakrabarti D, Wang Y, Wang C, Leskovec J, Faloutsos C. Epidemic thresholds in real networks. ACM Transactions on Information and System Security. 2008;10(4):1 doi: 10.1145/1284680.1284681

[13] Ferreira SC, Castellano C, Pastor-Satorras R. Epidemic thresholds of the susceptible infected susceptible model on networks: A comparison of numerical and theoretical results. Physical Review E. 2012;86(4):41–125. doi: 10.1103/PhysRevE.86.041125 10.1103/PhysRevE.86.041125 23214547

[14] Leskovec J, Krause A, Guestrin C, Faloutsos C, VanBriesen J, Glance N. Cost-effective outbreak detection in networks. In: Proceedings of the 13th ACM SIGKDD International Conference on Knowledge Discovery and Data Mining; 2007. p. 420–429.

[15] Shirazipourazad S, Bogard B, Vachhani H, Sen A, Horn P. Influence propagation in adversarial setting: how to defeat competition with least amount of investment. In: Proceedings of the 21st ACM International Conference on Information and knowledge management; 2012. p. 585–594.

[16] Kitsak M, Gallos LK, Havlin S, Liljeros F, Muchnik L, Stanley HE, et al Identification of influential spreaders in complex networks. Nature physics. 2010;6(11):888 doi: 10.1038/nphys1746

[17] Borge-Holthoefer J, Moreno Y. Absence of influential spreaders in rumor dynamics. Physical Review E. 2012;85(2):026116 doi: 10.1103/PhysRevE.85.026116 10.1103/PhysRevE.85.026116 22463288

[18] Borge-Holthoefer J, Rivero A, Moreno Y. Locating privileged spreaders on an online social network. Physical review E. 2012;85(6):066123 doi: 10.1103/PhysRevE.85.066123 10.1103/PhysRevE.85.066123 23005178

[19] Liu JG, Ren ZM, Guo Q. Ranking the spreading influence in complex networks. Physica A: Statistical Mechanics and its Applications. 2013;392(18):4154–4159. doi: 10.1016/j.physa.2013.04.037

[20] Nguyen NP, Yan G, Thai MT, Eidenbenz S. Containment of misinformation spread in online social networks. In: Proceedings of the 4th Annual ACM Web Science Conference; 2012. p. 213–222.

[21] Blondel VD, Guillaume JL, Lambiotte R, Lefebvre E. Fast unfolding of communities in large networks. Journal of statistical mechanics: Theory and experiment. 2008;2008(10):P10008 doi: 10.1088/1742-5468/2008/10/P10008

[22] Newman M, Ferrario CR. Interacting epidemics and coinfection on contact networks. PLOS ONE. 2013;8(8):e71321 doi: 10.1371/journal.pone.0071321 23951134 10.1371/journal.pone.0071321 PMC3738632

[23] Myers SA, Leskovec J. Clash of the Contagions: Cooperation and Competition in Information Diffusion. IEEE 12 International Conference on Data Mining. 2012;12:539–548.

[24] Wang W, Liu QH, Cai SM, Tang M, Braunstein LA, Stanley HE. Suppressing disease spreading by using information diffusion on multiplex networks. Scientific reports. 2016;6. 10.1038/srep29259 PMC4933956 27380881

[25] Sahneh FD, Scoglio C. Competitive epidemic spreading over arbitrary multilayer networks. Physical Review E. 2014;89:062817 doi: 10.1103/PhysRevE.89.062817 10.1103/PhysRevE.89.062817 25019843

[26] Wu Q, Lou Y, Zhu W. Epidemic outbreak for an SIS model in multiplex networks with immunization. Mathematical Biosciences. 2016;(277):38–46. doi: 10.1016/j.mbs.2016.04.004 27105863 10.1016/j.mbs.2016.04.004

[27] Kaur H, He JS. Blocking negative influential node set in social networks: from host perspective. Transactions on Emerging Telecommunications Technologies. 2015;

[28] Stich L, Golla G, Nanopoulos A. Modelling the spread of negative word-of-mouth in online social networks. Journal of Decision Systems. 2014;23(2):203–221. doi: 10.1080/12460125.2014.886494

[29] Perra N, Balcan D, Gonçalves B, Vespignani A. Towards a characterization of behavior-disease models. PLOS ONE. 2011;6(8):e23084 doi: 10.1371/journal.pone.0023084 21826228 10.1371/journal.pone.0023084 PMC3149628

[30] Wang Y, Xiao G, Wong L, Fu X, Ma S, Cheng TH. Effects of fear factors in disease propagation. Journal of Physics A: Mathematical and Theoretical. 2011;44(35):355101 doi: 10.1088/1751-8113/44/35/355101

[31] Deijfen M. Epidemics and vaccination on weighted graphs. Mathematical Biosciences. 2011;232(1):57–65. doi: 10.1016/j.mbs.2011.04.003 21536052 10.1016/j.mbs.2011.04.003

[32] Zhao S, Wu J, Ben-Arieh D. Modeling infection spread and behavioral change using spatial games. Health Systems. 2015;4(1):41–53. doi: 10.1057/hs.2014.22

[33] Kiss IZ, Cassell J, Recker M, Simon PL. The impact of information transmission on epidemic outbreaks. Mathematical Biosciences. 2010;225(1):1–10. doi: 10.1016/j.mbs.2009.11.009 19948177 10.1016/j.mbs.2009.11.009

[34] Li S, Zhu Y, Li D, Kim D, Huang H. Rumor restriction in online social networks. In: Proceedings of the 32nd IEEE International Performance Computing and Communications Conference; 2013. p. 1–10.

[35] Masuda N. Immunization of networks with community structure. New Journal of Physics. 2009;11(12):123018 doi: 10.1088/1367-2630/11/12/123018

[36] Kotnis B, Kuri J. Cost Effective Rumor Containment in Social Networks. Preprint Available from: arXiv:14036315. 2014;.

[37] Liontis K, Pitoura E. Boosting Nodes for Improving the Spread of Influence. Preprint Available from: arXiv:160903478. 2016;.

[38] Wang C, Chen W, Wang Y. Scalable influence maximization for independent cascade model in large-scale social networks. Data Mining and Knowledge Discovery. 2012;25(3):545–576. doi: 10.1007/s10618-012-0262-1

[39] Budak C, Agrawal D, El Abbadi A. Limiting the spread of misinformation in social networks. In: Proceedings of the 20th International Conference on World Wide Web. ACM; 2011. p. 665–674.

[40] Li HJ, Zhang C, Zhang XS. A study of inflammation immunization strategy in weighted complex network. In: Proceedings of the 11th IET Internation Symposium on Operations Research and its Applications in Engineering, Technology and Management; 2013. p. 1–7.

[41] Kimura M, Saito K, Motoda H. Minimizing the Spread of Contamination by Blocking Links in a Network. In: Proceedings of 23rd AAAI Conference on Artificial Intelligence; 2008. p. 1175–1180.

[42] Romero DM, Meeder B, Kleinberg J. Differences in the mechanics of information diffusion across topics: idioms, political hashtags, and complex contagion on twitter. In: Proceedings of the 20th international conference on World wide web; 2011. p. 695–704.

[43] Yadav A, Chan H, Xin Jiang A, Xu H, Rice E, Tambe M. Using social networks to aid homeless shelters: Dynamic influence maximization under uncertainty. In: Proceedings of the 2016 International Conference on Autonomous Agents & Multiagent Systems; 2016. p. 740–748.

[44] Wilder B, Yadav A, Immorlica N, Rice E, Tambe M. Uncharted but not Uninfluenced: Influence Maximization with an uncertain network. In: Proceedings of the 16th Conference on Autonomous Agents and MultiAgent Systems; 2017. p. 1305–1313.

[45] Chen D, Lü L, Shang MS, Zhang YC, Zhou T. Identifying influential nodes in complex networks. Physica a: Statistical mechanics and its applications. 2012;391(4):1777–1787. doi: 10.1016/j.physa.2011.09.017

[46] Albert R, Barabási AL. Statistical mechanics of complex networks. Reviews of modern physics. 2002;74(1):47 doi: 10.1103/RevModPhys.74.47

[47] Boccaletti S, Latora V, Moreno Y, Chavez M, Hwang DU. Complex networks: Structure and dynamics. Physics reports. 2006;424(4-5):175–308. doi: 10.1016/j.physrep.2005.10.009

[48] Watts D, Strogatz S. Collective dynamics of’small-world’networks. Nature. 1998;393(6684):440–442. doi: 10.1038/30918 9623998 10.1038/30918

[49] Barabási AL, Bonabeau E. Scale-free networks. Scientific American. 2003;288(5):50–59. 10.1038/scientificamerican0503-60 12701331

[50] Kleinberg JM. Navigation in a small world. Nature. 2000;406(6798):845 doi: 10.1038/35022643 10972276 10.1038/35022643

[51] Barabasi AL, Albert R. Emergence of Scaling in Random Networks. Science. 1999;286(5439):509–512. doi: 10.1126/science.286.5439.509 10521342 10.1126/science.286.5439.509

[52] Klemm K, Eguiluz VM. Growing scale-free networks with small-world behavior. Physical Review E. 2002;65(5):057102 doi: 10.1103/PhysRevE.65.057102 10.1103/PhysRevE.65.057102 12059755

[53] Cardillo A, Gómez-Gardenes J, Zanin M, Romance M, Papo D, Del Pozo F, et al Emergence of network features from multiplexity. Scientific reports. 2013;3:1344 doi: 10.1038/srep01344 23446838 10.1038/srep01344 PMC3583169

